# Development of the Esterase PestE for Amide Bond Synthesis Under Aqueous Conditions: Enzyme Cascades for Converting Waste PET into Tamibarotene

**DOI:** 10.1002/anie.202414162

**Published:** 2024-11-19

**Authors:** Ellie Goulding, Lucy C. Ward, Faye E. Allan, Drew Dittman, Jesus E. Salcedo‐Sora, Andrew J. Carnell

**Affiliations:** ^1^ Department of Chemistry University of Liverpool Crown Street Liverpool L69 7ZD United Kingdom; ^2^ GeneMill Institute of Integrative Biology University of Liverpool Crown Street Liverpool L69 7ZB United Kingdom

**Keywords:** Biocatalysis, amidation, enzymes, synthetic methods, polymers

## Abstract

A growing number of hydrolase enzymes show promiscuous acyltransferase activity, even under aqueous conditions. Here we report, for the first time, the ability of *Pyrobaculum calidifontis* VA1 esterase (PestE) to catalyse the formation of a wide range of amides in buffer, where the acyl donor forms a significant structural component in the amide product. The reactions occur under mild conditions and can achieve conversions up to 97 % in 6 h for formation of N‐benzylfuranamide as the model reaction. We demonstrate PestE's potential in enzyme cascades to make amides from waste PET plastic and the conversion of the terephthalic acid product to tamibarotene, a drug with activity against acute leukemia. Rational mutagenesis led to identification of PestE variants F33L F289A and F33L. F33L F289A increased conversion of *N*‐benzylfuranamide by 1.2‐fold, and F33L gave a 4‐fold increase in conversion to tamibarotene.

## Graphical abstract

The esterase PestE shows promiscuous activity in catalysing the formation of a wide range of amides under aqueous conditions and can be integrated into enzyme cascades as demonstrated in the conversion of polyethylene terephthalate (PET) degradation product terephthalate into the leukemia drug tamibarotene. PestE variants F33L F289A and F33L show improved activity for a model reaction and tamibarotene formation respectively.

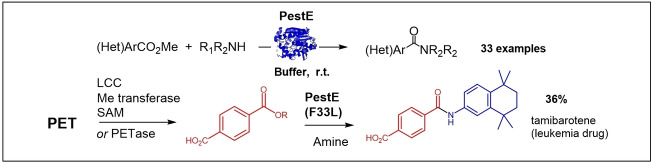

## Introduction

Amides are important functional groups in many biologically active compounds and pharmaceutical drugs, with 25 % of all pharmaceuticals containing an amide bond.^[1]^ Amide bond formation has been recognised as the most frequently used reaction in the industrial synthesis of pharmaceuticals, constituting 16 % of all reactions.^[1]^ Carboxylic acids are generally activated as acid chlorides or anhydrides prior to reaction with amines. Alternatively, a wide range of coupling reagents such as DCC (dicyclohexylcarbodiimide), HOBt (1‐hydroxybenzotriazole) and HATU (hexafluorophosphate azabenzotriazole tetramethyl uronium) can be used.^[2]^ However, these approaches use toxic materials or require difficult separations and are not atom economic. Indeed, improving the atom economy of amide bond formation was recently identified as an urgent priority for greener, sustainable synthesis.^[3]^ Biocatalytic methods have emerged as a complementary approach with lipases most often used for the synthesis of esters and also amides, although the use of organic solvents to minimise competing hydrolysis reduces sustainability.^[4]^ The ability to catalyse acyl transfer reactions to synthesise amides in aqueous media is extremely desirable. Only a few examples of hydrolases able to catalyse ester or amide bond formation in bulk water have been identified. For many years penicillin acylase has been used to catalyse the kinetically controlled synthesis of β‐lactam antibiotics.^[5]^ Lipases/acyltransferases such as CpLip2 from *Candida parapsilosis* and CalAc5 from *C. albicans* were shown to catalyse the transesterification of oleate esters in buffer with a kinetic preference for the acyl transfer.^[6]^ These enzymes produced transient high levels of ester products before hydrolysis of the product to give the carboxylic acids. An acyltransferase from *Mycobacterium smegmatis* (MsAcT) was originally shown to catalyse transesterification under aqueous conditions.[^7^, ^8^, ^9^, ^10^, ^11^, ^12^] It is one of few promiscuous acyltransferases able to catalyse amide formation, with Land *et al*. first reporting this in a one‐pot cascade reaction where a transaminase enzyme was used to form an amine from an aldehyde/ketone prior to acylation, giving the amide in 92 % yield.^[13]^ Paradisi *et al*. then expanded the substrate scope of MsAcT to include several primary aromatic amines for the production of amides in excellent yields.^[14]^ However, a large excess of vinyl acetate or ethyl acetate was often required and reactions needed to be carefully monitored and stopped prior to hydrolysis of the product. Amide hydrolysis has recently been addressed by conducting MsAcT reactions with immobilised enzyme in a continuous flow reactor. Using 0.25–0.5 M amine and a liquid‐liquid phase of buffer/EtOAc 90 : 10, continuous product removal avoided any amide hydrolysis.^[15]^ A serine to cysteine mutation at the catalytic serine of MsAcT allowed further expansion of the substrate scope to include secondary amines and thiols as acyl acceptors.^[16]^ Further mutagenesis of MsAcT enabled expansion of the active site to accommodate longer acyl chains (up to octanoyl) in transesterifications, with improvements in acyl transfer to hydrolysis ratios.^[8]^ A *C*‐acyltransferase, PpATaseCH, from *Pseudomonas protegens* has also been shown to catalyse the *N*‐acetamidation of a range of hydroxylaniline derivatives in water, achieving up to 96 % yield when 1.5 equivalents of phenyl acetate were used as the acyl donor.^[17]^

Bornscheuer reported the promiscuous acyltransferase activity of family VIII carboxylesterases, with an acyl transfer to hydrolysis ratio higher than that obtained with MsAcT.^[18]^ The best enzyme, EstCE1 was found to catalyse acyl transfer to benzyl alcohol or benzylamine using an excess of simple acyl donors or dimethyl carbonate respectively. There are only few reported examples of amide formation from a non‐simple ester substrate, including EstCE1, which catalysed the formation of pharmaceutical drug moclobemide (from methyl 4‐chlorobenzoate and 4‐(2‐aminoethyl)morpholine), albeit in only 20 % yield. MsAcT has also been demonstrated to catalyse formation of vanillamides in modest yields of 15–40 %.^[19]^ Lipase SpL (from *Sphingomonas* sp. HXN‐200) has very recently been reported for the *N*‐acylation of ethyl esters with aromatic and aliphatic amines in buffer, on preparative scales.^[20]^

A sequence‐based prediction method enabled prediction of 5 members of the bacterial hormone sensitive lipase family (bHSL) with high acyl transferase activity in buffer.^[21]^ This activity was shown to correlate closely with hydrophobicity of the substrate binding pocket. One of the most efficient was *Pyrobaculum calidifontis* VA1 esterase (PestE) (PDB code: 3ZWQ), first identified by Hotta *et al*. as a highly thermostable enzyme.^[22]^ Rational mutagenesis then gave variants with enhanced activity and selectivity for formation of a range of terpene acetates in water using ethyl acetate as the acyl donor.^[23]^ PestE had been largely unexplored for amide formation until very recently, where it has been shown to catalyse the formation of a range of carbamates using divinyl carbonate as acyl donor.^[24]^ Also, a PestE triple mutant was able to catalyse formation of hydroxycinnamic acid amides starting from the vinyl or methyl ester and several amines.^[25]^

ATP‐dependent carboxylic acid reductases are able to catalyse the formation of a range of amides from carboxylic acids and amines via an adenylate intermediate. Several pharmaceutically relevant monoacylated diamines were produced in high yields, although required over 100‐fold excess of amine.^[26]^ These enzymes have also recently been applied to lactam formation and surfactant formation.^[27]^ Other examples include non‐ribosomal peptide synthases (NRPSs) and amide bond ligases.^[28]^ However, these enzymes require an activated substrate such as an acyl‐coenzymeA (acyl‐CoA) ester or have a high selectivity for specific acyl donors and acceptors, limiting their application as isolated biocatalysts.^[29]^ Another ATP‐dependent enzyme, amide bond synthetase McbA, catalysed the formation of moclobemide in >99 % conversion, using 1.5 equivalents of amine although with low substrate loadings.[^30^, ^31^]

**Scheme 1:**
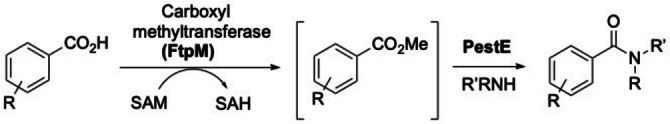
Proposed enzymatic cascade using carboxyl methyltransferase (FtpM) to activate a carboxylic acid for *in situ* coupling with amines to form amides in buffer, catalysed by esterase PestE. FtpM catalysed methylation requires co‐factor SAM (S‐adenosyl methionine) as methyl donor.

We now show that PestE can catalyse the formation of a range of synthetically useful amides, including tertiary amides, in water using as little as 2 : 1; amine:ester ratio. Substrates include heteroaromatic and substituted benzoic acid methyl esters, aniline, benzylamines and butylamine, one of the broadest substrate scopes for a hydrolase/acyltransferase described. This is the first demonstration of the potential of this enzyme for making amides where both components are significant structural components rather than simple acyl or carbamate derivatives and moves away from using excess of simple acyl donors such as vinyl or ethyl acetate, as is the case for most current literature examples. Enzymes that have been applied to bulkier acyl donors have limited conversions *i.e*. 15–40 % vanillamide conversion^[19]^ with MsAcT and 20 % moclobemide with EstCE1.^[18]^ The ability to conduct amidation reactions in water facilitates cascades with other enzymes. For example, we have recently developed a carboxyl methyltransferase (FtpM) for the mono and dimethylation of carboxylic acids such as terephthalic acid (TA) and 2,5‐furandicarboxylic acid (FDCA).^[32]^ Thus enzyme cascades for coupling of carboxylic acids with amines via the intermediacy of an enzyme catalysed methyl ester becomes possible (Scheme 1). In fact, this is a strategy used by nature in the biosynthesis of capuramycin‐type antibiotic compounds from carboxylic acids by methyltransferase CapS and *N*‐acyltransferase CapW.[^33^, ^34^]

In this paper we also demonstrate the use of PestE as part of an enzymatic cascade for conversion of waste polyethylene terephthalate (PET) plastic into the pharmaceutical drug tamibarotene, an orally active synthetic retinoid, developed to overcome all‐trans retinoic acid (ATRA) resistance with potential antineoplastic activity against acute promyelocytic leukemia.^[35]^ We also report the mutagenesis of PestE based on several strategies to increase amide formation and reduce acyl donor hydrolysis. The best mutant showed an improved rate of acyl transfer and increased tamibarotene conversion.

## Results and Discussion

### Reaction Optimisation of PestE Catalysed N‐acylation

**Figure 1:**
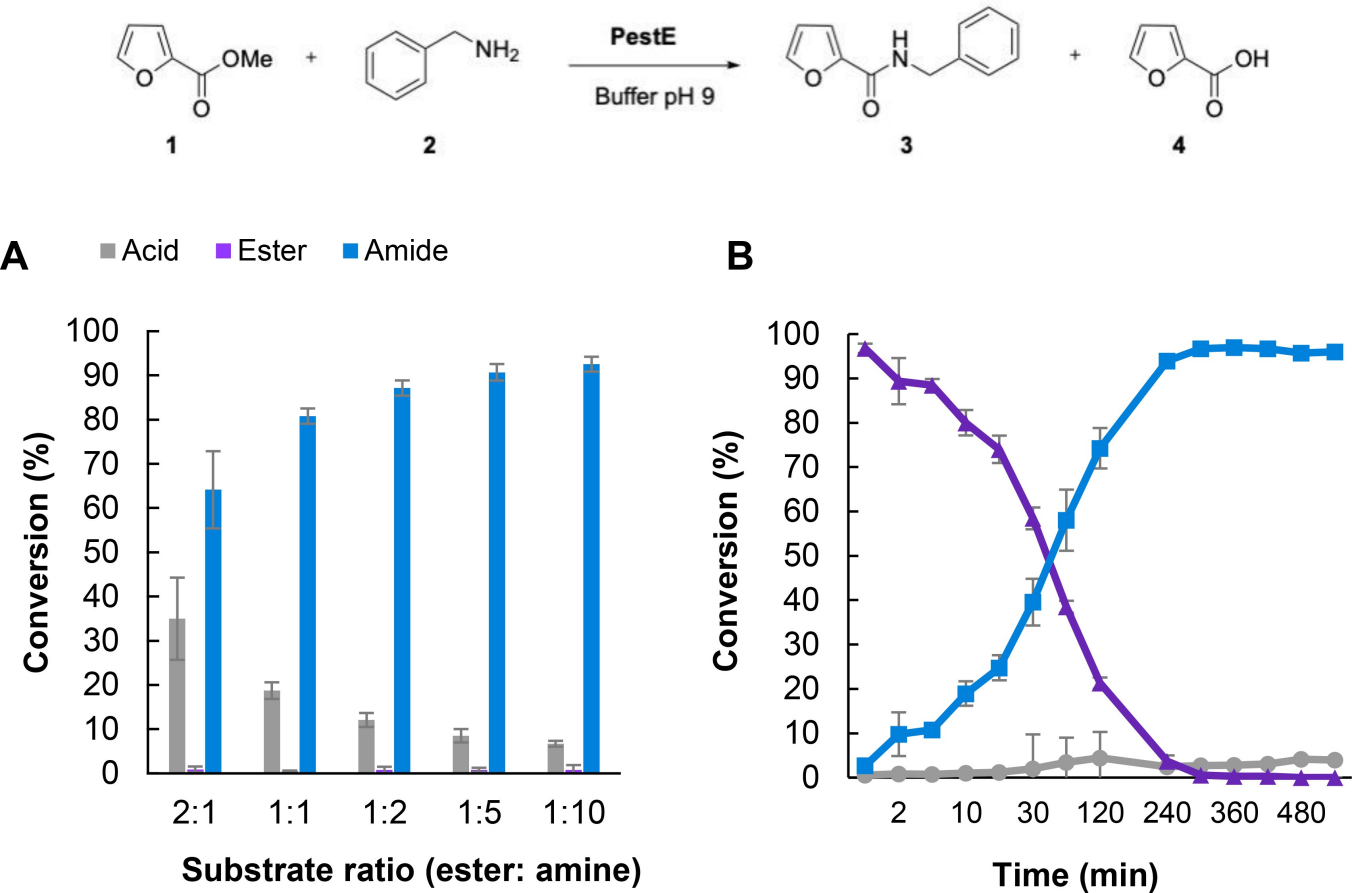
**A**: Varying amine concentration for PestE catalysed *N*‐acylation of benzylamine **2** with methyl 2‐furanoate **1**. Reaction consisted of methyl 2‐furanoate (10 mM), benzylamine hydrochloride (X mM) and PestE (100 μM) in 200 mM Tris buffer (pH 9) incubated for 18 h at 25 °C, 200 rpm. **B**: Time course reaction for PestE catalysed *N*‐benzylfuranamide **3** formation. Reaction consisted of methyl 2‐furanoate (10 mM), benzylamine hydrochloride (100 mM) and PestE (100 μM) in 200 mM Tris buffer (pH 9) incubated for 18 h at 25 °C, 200 rpm.

We cloned the gene encoding PestE and expressed the recombinant protein, before initially screening it along with several other hydrolases for amide bond forming activity (Figure S2). We observed good conversions to amide for model substrate methyl furanote **1** (10 mM) with benzylamine HCl (10 mM) as the corresponding amide **3** was formed with 48 % conversion. This was compared to hydrolases Cal‐A and Cal‐B which catalysed only hydrolysis of the acyl donor. We examined the effect of varying the ratio of furan ester **1** to amine on acyl transfer versus hydrolysis, and a clear preference for amide bond formation over hydrolysis was observed over the full range of ratios after 18 h. (Figure 1A). A ten‐fold excess of benzylamine **2** gave highest conversion to amide **3** (93 %) with minimal hydrolysis to **4** (7 %). No spontaneous amide formation or subsequent amide hydrolysis was observed over 24 h. Importantly, up to 10 % background hydrolysis of the acyl donor was observed without enzyme present, indicating some hydrolysis is non‐enzymatic.

The reaction between benzylamine **2** and methyl 2‐furanoate **1** was optimised for substrate concentration, pH, temperature and enzyme loading. Under the optimum conditions (10 mM ester, 100 mM amine HCl, 100 μM enzyme, pH 9.0), maximum conversion (93 %) to *N*‐benzylfuranamide **3** could be achieved in 6 h with minimal hydrolysis (Figure 1B). Lower amounts of amine and enzyme loadings could also be used (Table S3). Notably, 59 % conversion to amide was observed with just 2 eq of amine and 5 μM enzyme. PestE was also able to achieve high conversion starting with higher ester concentration (25 mM) and 2 eq. of amine (see preparative scale reactions). Importantly for future scale up and potential for screening, we also observed a reasonable 83 % conversion to amide under these optimised conditions using cell lysate.

### Amine Substrate Scope

We next examined the substrate scope by varying firstly the amine component with furanoate ester **1** and secondly the ester component with benzylamine **2**. Table 1 shows the range of amines that were coupled with methyl 2‐furanoate **1** using PestE under our optimised conditions, to give amide conversions ranging from 13–93 %. Most of the benzylamines gave good to excellent amide conversions, with little correlation between substitution and conversion. Gratifyingly, secondary benzylic amines **19** and **20** were also substrates, although selected secondary cyclic amines, diamines and sulphonyl anilines were not accepted (Table S4). The only other example of formation of tertiary amides by acyltransferases was with engineered MsAcT, and recently with PestE for carbamate formation.[^16^, ^32^] Several anilines also gave amide products. The lower nucleophilicity of aniline has meant anilines have scarcely been reported for amide formation with hydrolases/acyltransferases, so 78 % conversion with PestE to amides **32** and **33** was particularly interesting. Pleasingly, the aliphatic butylamine also gave a good (78 %) conversion to the amide **38**.

**Table 1:** PestE catalysed *N*‐acylation of methyl 2‐furanoate **1** with different amines. Conditions: 100 μM PestE, 10 mM ester, 100 mM amine HCl, 200 mM Tris buffer pH 9, 25 °C, 200 rpm, 18 h.

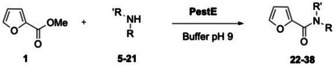		
Amine substrate	Acid (% conv. ) (**4**)	Amide (% conv.) (**X**)
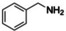	7	93 (**3**)
2		
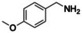	46	53 (**22**)
5		
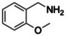	7	93 (**23**)
6		
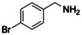	14	30 (**24**)
7		
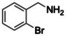	16	84 (**25)**
8		
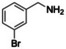	7	93 (**26**)
9		
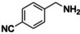	13	82 (**27**)
10		
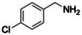	30	28 (**28**)
11		
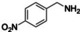	14	70 (**29**)
12		
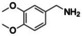	42	58 (**30**)
13		
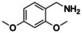	13	86 (**31**)
14		
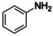	22	78 (**32**)
15		
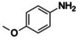	19	78 (**33**)
16		
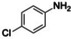	46	43 (**34**)
17		
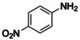	42	56 (**35**)
18		
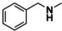	85	13 (**36**)
19		
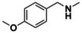	68	31 (**37**)
20		
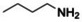	22	78 (**38**)
21		

In all bioconversions, acid formation is due to donor ester hydrolysis, as no amide hydrolysis was observed within the reaction time. Donor ester hydrolysis is also predominantly enzyme catalysed, with only low levels (<10 %) of non‐enzymatic hydrolysis observed in the absence of the enzyme. 2‐Furoic acid **4** formation is variable between amine substrates, as hydrolysis and amidation are competing, so less efficient amide formation allows the ester to be available for hydrolysis. Clearly with many of the best substrates, acyl transfer for amidation successfully out competes the rate of acyl donor hydrolysis. Notably, no amide products were formed in the absence of the enzyme for any substrates (Figures S8–S13).

### Ester Substrate Scope

Having demonstrated acceptance of a range of amine nucleophiles, we then explored the scope of esters with benzylamine under our optimised conditions (Table 2). The 2‐furanyl and 2‐thiophenyl esters **1** and **40** were converted to amides with similar high efficiency whereas the 3‐furanyl ester **39** amidation was significantly lower (42 %). In the methyl benzoate series, the parent ester **41** and 4‐acetyl ester **50** gave the highest amide conversions. The nitro substitution in **47** and **48** clearly slowed the rate of reaction significantly as did 3‐hydroxy and 3‐methoxybenzoates (**46** and **44**). However, other substitution was well tolerated in esters **50–54**. Of the esters tested, only monomethyl isophthalate **55** and ortho‐substituted benzoates **42** and **49** were not accepted as substrates, with only partial hydrolysis for ester **42** observed after 18 h.

**Table 2:** PestE catalysed *N*‐acylation of different methyl esters with benzylamine HCl **2**. Conditions: 100 μM PestE, 10 mM ester, 100 mM amine HCl, 200 mM Tris buffer pH 9, 25 °C, 200 rpm, 18 h.

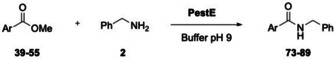		
Ester substrate	Acid ( % conv.) (**X**)	Amide ( % conv.) (**X**)
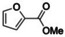	7 (**4**)	93 (**3**)
1		
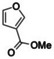	5 (**56**)	42 (**73**)
39		
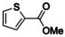	– (**57**)	91 (**74**)
40		
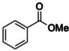	3 (**58**)	81 (**75**)
41		
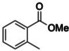	31 (**59**)	– (**76**)
42		
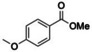	6 (**60**)	73 (**77**)
43		
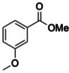	18 (**61**)	8 (**78**)
44		
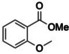	34 (**62**)	16 (**79**)
45		
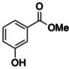	8 (**63**)	1 (**80**)
46		
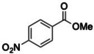	33 (**64**)	50 (**81**)
47		
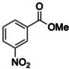	42 (**65**)	43 (**82**)
48		
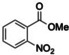	12 (**66**)	– (**83**)
49		
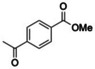	18 (**67**)	81 (**84**)
50		
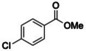	14 (**68**)	58 (**85**)
51		
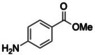	2 (**69**)	77 (**86**)
52		
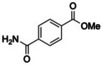	30 (**70**)	70 (**87**)
53		
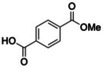	24 (**71**)	69 (**88**)
54		
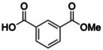	13 (**72**)	– (**89**)
55		

### 3‐Step Enzymatic Cascade from Waste Plastics to Amides

**Scheme 2:**
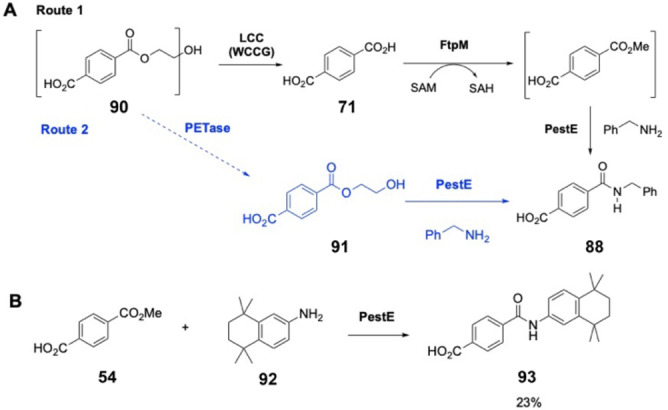
**A**: Enzymatic cascade routes to amides from PET. **Route 1**: 3‐step enzymatic cascade to amide **88** via LCC (WCCG), FtpM and PestE. **Route 2**: 2‐step enzymatic cascade to amide **88** via PETase and PestE. **B**: PestE catalysed amidation of MMT to form tamibarotene **93**, 23 % conversion. Conditions: 100 μM PestE, 5 mM ester, 10 mM amine, 200 mM NaPi buffer pH 8, 25 °C, 200 rpm, 18 h.

We were particularly pleased that monomethyl terephthalate (MMT) **54** was accepted to give a 69 % conversion to the amide **88** since we have previously developed a novel methyltransferase (FtpM) that can efficiently and selectively monomethylate PET monomer terephthalic acid (TA) **71** to MMT **54**.^[32]^ Thus, we envisaged an enzyme cascade for the conversion of waste post‐consumer PET plastic to high value amide products (Scheme 2A Route 1). The valorisation or upcycling of waste plastic has drawn particular attention recently.^[36]^ Indeed, Sadler *et al*. demonstrated the use of leaf branch compost cutinase mutant LCC (WCCG) for PET degradation followed by conversion of TA to products such as vanillin, using an engineered *E. coli* strain.[^37^, ^38^]

We used powdered PET from a post‐consumer PET bottle as a substrate for degradation by LCC (WCCG) over 48 h at 72 °C, pH 9.0 (Scheme 2A). After spinning off the residual unreacted polymer, the TA **71** concentration in solution was measured as 1.56 mM. Although not optimised, this was sufficient for us to demonstrate our proposed cascade to synthetically useful amides. After heat inactivation of the LCC (WCCG) at 100 °C, our previously developed carboxylmethyltransferase FtpM was added. During the PET degradation, the pH decreased to 8.0 due to the TA formation, providing appropriate conditions for the subsequent FtpM‐catalysed methylation. We were pleased to observe quantitative conversion to the monomethyl ester, MMT **54** (1.54 mM). We then added PestE to catalyse the final amide coupling step (Scheme 2A Route 1). Benzylamine was initially coupled to MMT to give the corresponding amide **88**, albeit with just 7 % conversion. This is much lower than the reaction in isolation (69 %) and suggests that the ester concentration in the cascade, resulting from the limitation in the LCC reaction, is too low for PestE. As an alternative strategy we also tested 2‐hydroxyethylterephthalic acid (MHET) **91** as a substrate for PestE, replacing MMT (Scheme 2A Route 2). MHET is the major degradation product produced from PET by PETase and thus this would bypass the requirement for the FtpM step. To our delight, PestE was able to convert MHET into the corresponding benzylamide **88** in 25 % conversion.

We then identified the drug tamibarotene **93** as a valuable synthetic target. Tamibarotene is a synthetic retinoid that has recently shown promising activity against acute promyelocytic leukemia (APL).^[35]^ It has been approved in Japan as a chemotherapy treatment for APL patients. The drug has also been investigated as a possible treatment for myelodysplastic syndrome, Alzheimer's disease, multiple myeloma, Crohn's disease and SARS‐CoV2 (Severe Acute Respiratory Syndrome Coronavirus 2).[^35^, ^39^, ^40^, ^41^] The published large‐scale synthesis involves coupling the acid chloride of monomethyl terephthalate with the amine **92** followed by deprotection of the methyl ester.^[42]^ Production of tamibarotene from waste PET **90** or even terephthalic acid **71** itself in our enzymatic cascade would preclude the need for acid chloride formation and the deprotection step. We were delighted to observe that the commercially available amine **92** was coupled with MMT **54** using PestE to give 23 % conversion to tamibarotene **93** (Scheme 2B). 62 % conversion to TA was also observed, along with 15 % unreacted ester remaining. It was necessary to use vigorous mixing by vortexing in order to solubilise the highly hydrophobic amine, which may explain the modest yield. Unfortunately, no conversion to tamibarotene was observed when starting from PET via our 3‐step enzymatic cascade, which again could be attributed to the low product concentration from the LCC (WCCG) plus methylation steps (1.54 mM) being insufficient to allow PestE to catalyse amidation.

We also demonstrated potential for a 2‐step enzymatic cascade to produce tamibarotene and other pharmaceutically relevant amides from carboxylic acids, using FtpM and PestE (Table S8). Selective monoamidation of symmetric diacids without the need for protection steps is synthetically attractive, and the pendant acid group allows for further functionalisation to more complex compounds.

### Engineering of PestE

**Figure 2:**
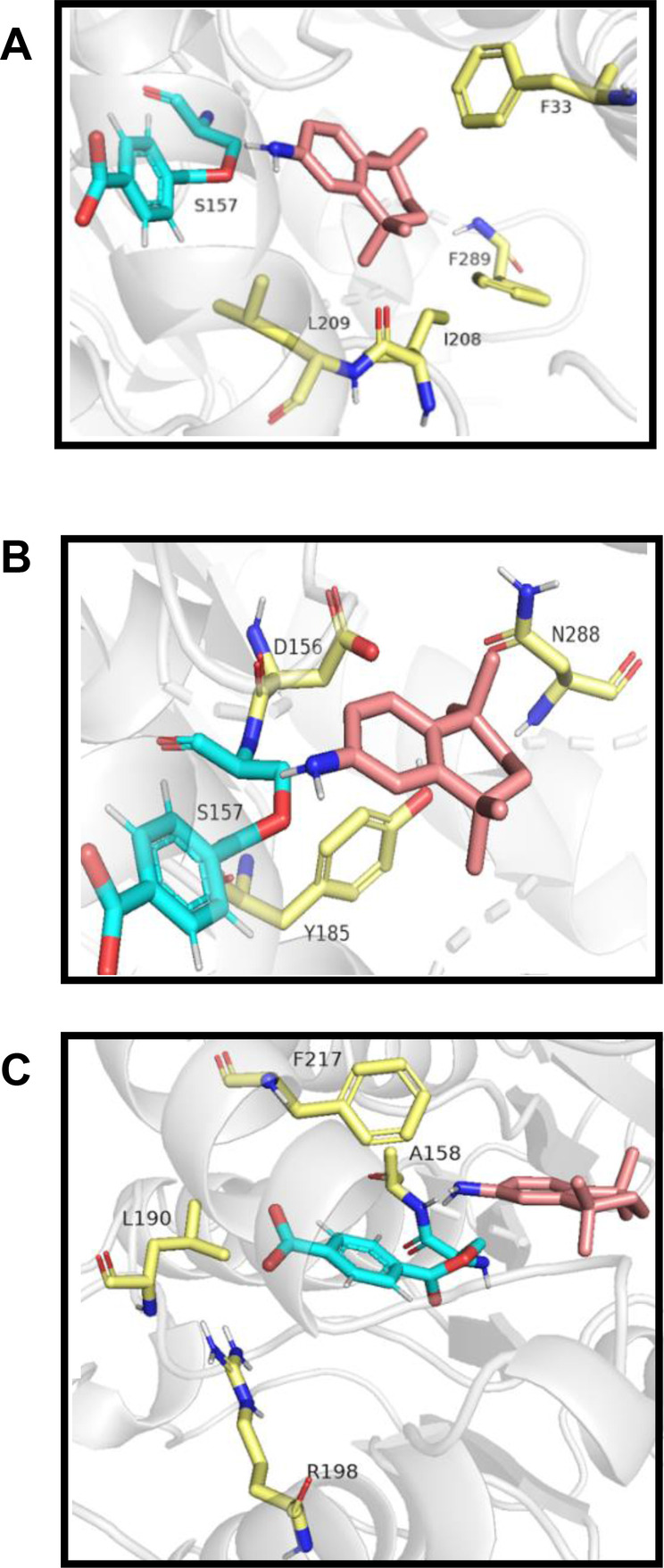
Docking of amine **92** in active site of acylated PestE (PBD: 3ZWQ) structure. Residues selected based on (A) large residues in amine binding tunnel, (B) residues involved in coordinating water, and (C) residues that may be involved in stabilising the acyl enzyme intermediate. Blue: TA acylated catalytic serine 157, pink: amine **92**, yellow: possible residues for mutation.

We hoped engineering of PestE would improve the formation of tamibarotene from MMT **54** and therefore allow production from waste PET, as well as improving general *N*‐acylation activity. In order to identify residues to target for mutagenesis, the amine moiety of tamibarotene was docked into the active site of the acylated PestE crystal structure using Webina (Figure 2).^[43]^

Residues within 4 Angstroms of the docked amine **92** were selected based on 4 strategies for improving acyltransferase activity that have been successful in other studies. These include increasing the size of the acyl acceptor tunnel, disruption of the network of amino acids that bind water in the active site, stabilising the acyl‐enzyme intermediate and increasing hydrophobicity of active site residues. Figure 2A–C displays amino acids that could target each of the first 3 strategies respectively.[^8^, ^23^, ^44^, ^45^] Mutants F33L and F289A were selected to increase the size of the amine binding tunnel, to better accommodate the bulky amine (Figure 2A). Y185F was selected to disrupt the water network (Figure 2B) as Y185 is involved in hydrogen bonding to water molecules to facilitate nucleophilic attack. F217A was selected as it could have an effect on enzyme intermediate stability, and stabilising the acyl enzyme intermediate has been proven to increase amidation activity significantly more than hydrolysis activity, due to the higher nucleophilicity of amines over water (Figure 2C). Generally, each mutant was selected to either maintain or increase hydrophobicity, as the correlation between acyltransferase activity and hydrophobicity of active site residues has been previously described.[^21^, ^44^] S157C was selected due to the previous success of a Ser to Cys variant of MsAcT produced by Paradisi *et al*., to allow expansion of substrates to secondary amines and thiols.^[16]^

**Figure 3:**
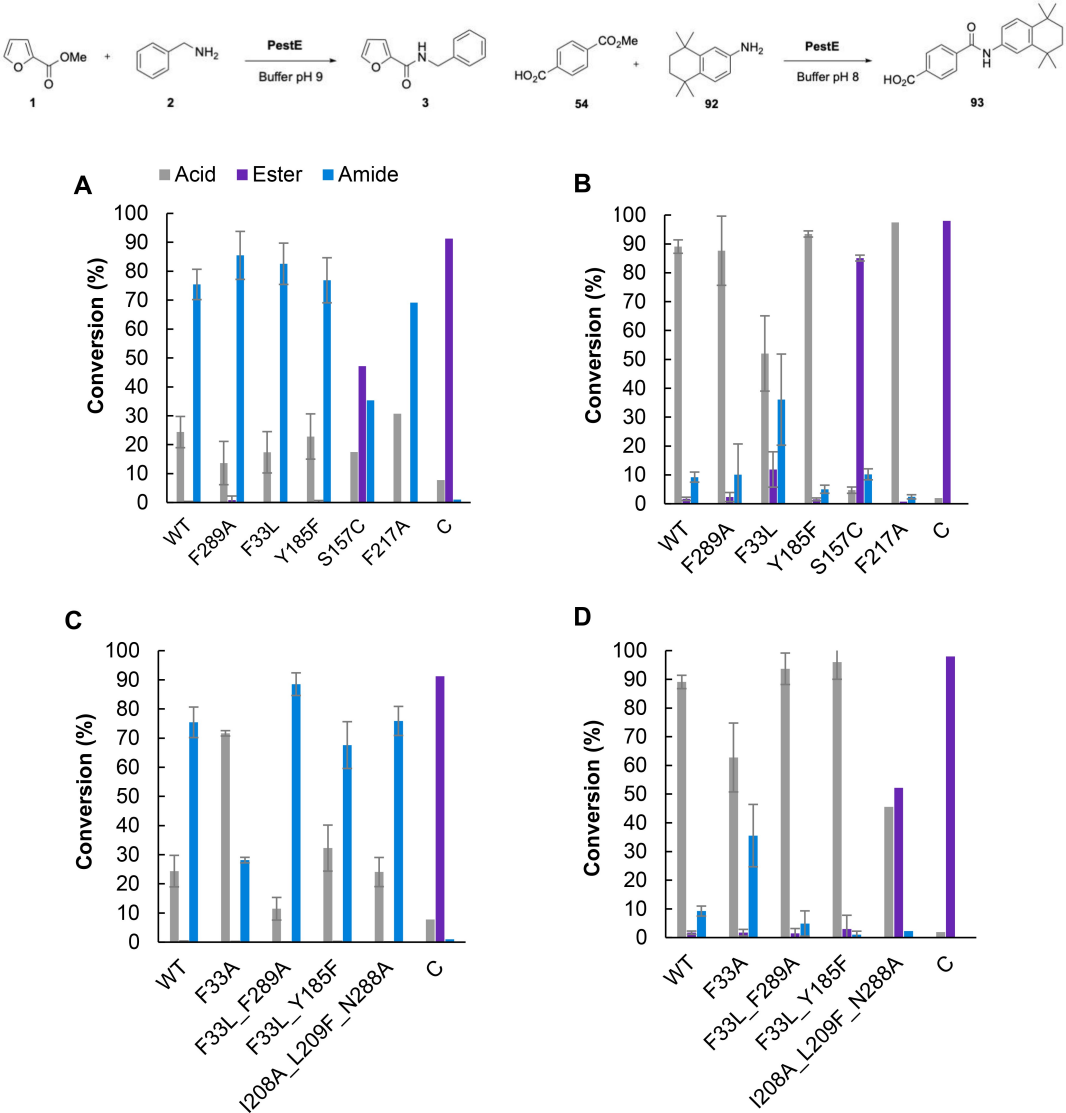
A and C: Formation of *N*‐benzylfuranamide **3** by PestE mutants. Acid: 2‐furoic acid, ester: methyl 2‐furanoate, amide: *N*‐benzylfuranamide. Conditions: 100 μM PestE, 10 mM ester, 10 mM amine HCl, 200 mM Tris buffer pH 9, 25 °C, 200 rpm, 18 h. B and D: Formation of tamibarotene **93** by PestE mutants. Acid: terephthalic acid, ester: monomethyl terephthalate, amide: tamibarotene. Conditions: 100 μM PestE, 5 mM ester, 10 mM amine, 200 mM NaPi buffer pH 8, 25 °C, 200 rpm, 18 h. C: no‐enzyme (reaction set up as before but with no enzyme and storage buffer replacing the enzyme).

Mutants were selected and produced as for wild type PestE, with no significant effects on protein yield (Table S1). In addition to tamibarotene **93** formation (Figure 3B) the mutants were also simultaneously tested for the model reaction (N‐benzylfuranamide formation, Figure 3A). For the model reaction (1 : 1 ester: amine), mutants F289A and F33L improved amide formation by up to 10 % (85 % and 83 % respectively). This indicates increasing tunnel size was an efficient strategy. Y185F gave comparable amide conversion to WT, suggesting a non‐essential role for the phenolic OH in the side chain. Mutant S157C showed minimal acyl transfer and hydrolysis activity, indicating the importance of S157 as a catalytic residue.

Interestingly, for the tamibarotene **93** forming reaction (Figure 3B; 2 : 1 amine : ester), F33L afforded a significant increase in amide formation (36 %)‐ 4‐fold higher than WT when testing the *N*‐acylation of MMT **54**, whereas F289A showed no improvement. This can be attributed to the close proximity of the F33L side chain to the bulky methyl groups on the amine **92** (Figure 2A). Thus, changing the steric demands whilst retaining hydrophobicity may provide better access of the tamibarotene amine **92** to the acylated serine.

Based on these results, iterative mutagenesis was performed to produce double mutants F33L F289A and F33L Y185F. As mutants F33L and F289A were beneficial for both tamibarotene formation and the model reaction respectively, it was hoped mutant F33L F289A would increase the amide conversion of both reactions by increasing the tunnel size further at 2 positions. As mutant Y185F also performed well for the model reaction, mutant F33L Y185F was produced to determine if combining the strategies of increasing tunnel size with disruption of the water network would further improve amide conversion. Due to the success of F33L mutant with increasing tamibarotene conversion up to 36 %, mutant F33A was produced in the hope that reducing the residue size would further improve tamibarotene conversion. Triple mutant PestE I208A I209L N288A was reported by Bornscheuer *et al*. for hydroxycinnamide formation and to have significantly improved acyltransferase activity for the acetylation of hydroxytyrosol, so was also included in this study.[^25^, ^45^]

**Figure 4:**
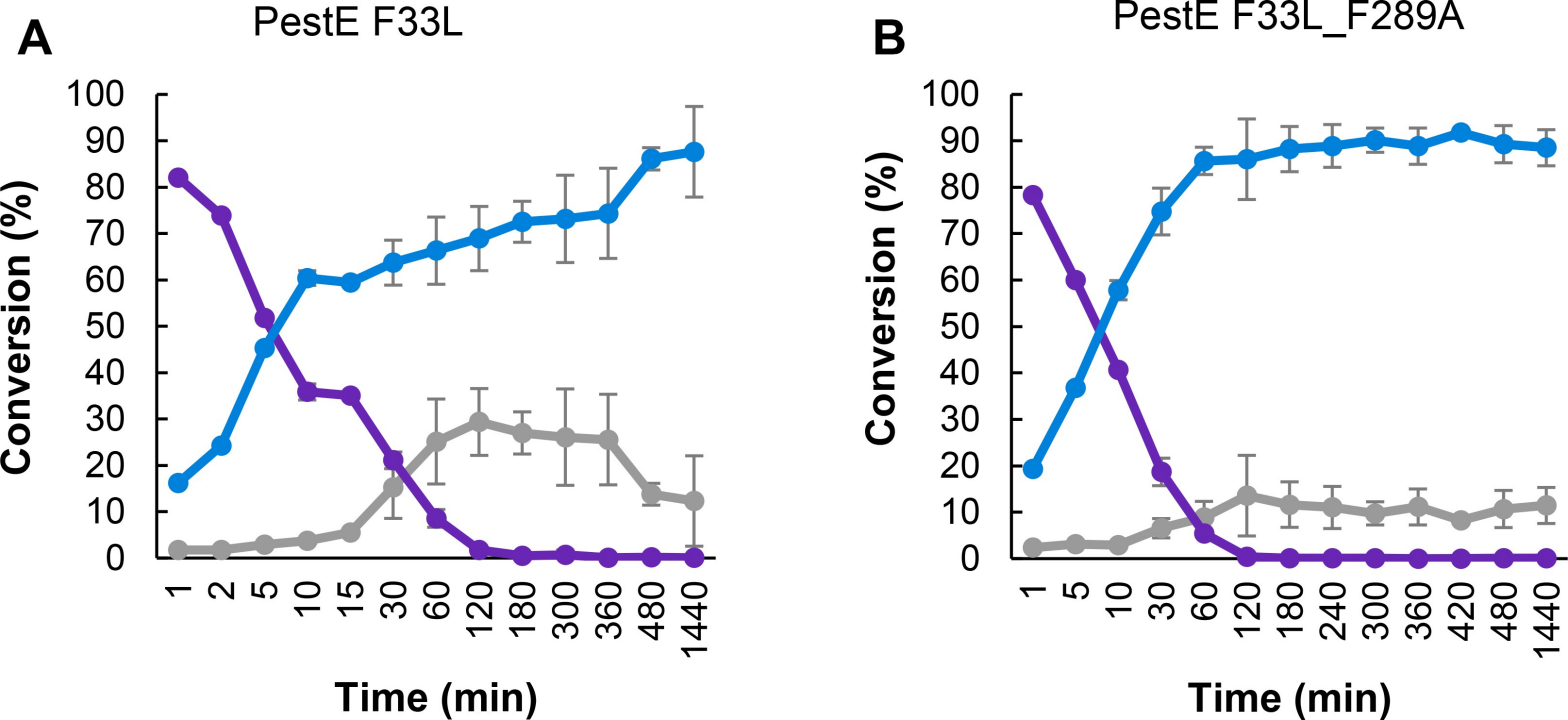
Time course reactions for *N*‐benzylfuranamide formation from methyl 2‐furanoate and benzylamine HCl, catalysed by **A** PestE F33L and **B** PestE F33L F289. Acid: 2‐furoic acid (grey), ester: methyl 2‐furanoate (purple), amide: N‐benzylfuranamide (blue). Conditions: 100 μM PestE, 10 mM methyl 2‐furanoate, 10 mM benzylamine HCl, 200 mM Tris buffer pH 9, 25 °C, 200 rpm, 18 h.

From the second batch of mutants, F33L F289A gave a 1.2‐fold increase in furanamide **3** conversion compared to the WT after 18 h (Figure 3C). Time course studies were carried out with both variants F33L and F33L F289A for the model reaction to gain a clearer understanding of how it proceeds (Figure 4). This confirmed that both variants had higher rates of amide formation than the WT, even using 1 : 1 amine:ester compared the 10 : 1 for the WT (Figure 4 cf. Figure 1B)). Furthermore, a synergistic effect of these mutations appears to diminish the hydrolysis markedly, (Cf Figures 4 A and B) allowing a much faster kinetic accumulation of the amide. The double mutant F33L F289A catalyses 85 % conversion to furanamide **3** after 60 min compared with 480 minutes for F33L. For tamibarotene formation, F33A (Figure 3C) maintained the higher conversion to tamibarotene **93** (36 %) obtained with F33L (Figure 3D).

### Preparative Scale Reactions

As representative examples to demonstrate potential for scale up, PestE catalysed reactions for the formation of *N*‐benzylfuranamide **3** and tamibarotene **93** were carried out on a 10 mL and 15 mL scale respectively. With 25 mM ester substrate loading and a 2‐fold amine excess, isolated yields of 62 % (31 mg) and 72 % (36 mg) of *N*‐benzylfuranamide were achieved using WT PestE and mutant F33L F289A respectively, with no purification step required (ESI). Prior to extraction, the reaction conversions were 75 % and 89 % respectively so the isolated yields are congruous with conversions obtained. Using 25 mM MMT **54** and 50 mM amine **92**, 19 mg of tamibarotene **93** was isolated (15 % yield) using PestE mutant F33A (ESI). For tamibarotene formation, the conversion was maintained on scale up and the lower isolated yield is attributed to product losses during purification. Both amide products were fully characterised. This demonstrates that the biotransformation has potential to produce pharmaceutically relevant amides on scale.

## Conclusions

We have successfully demonstrated the *N*‐acylation activity of acyltransferase PestE for a range of ester and amine substrates. PestE is capable of catalysing formation of a wide range of secondary and tertiary furanamides with 13–93 % conversions using substituted benzylamines, anilines and n‐butylamine. The model reaction, *N*‐benzylfuranamide **3** formation was performed on a preparative scale and the amide isolated without significant loss of product.

In addition, good conversions were observed for 2‐thiophenyl ester and selected benzoate esters with benzylamine as the model amine (up to 91 % amide). Most literature examples of acyltransferases utilise simple acetate donors as sacrificial esters, in a large excess (20×) to drive the reaction and provide a sufficient supply of the ester. There are very few literature examples of forming amides using more synthetically useful acyl donors (non‐acetamides). Notable examples include MsAcT for the formation of vanillin analogues and EstCE1 for the formation of moclobemide. Although they require different conditions: 2‐fold excess of amine and 4‐fold excess of ester respectively, only modest yields of 15–40 % were achieved.[^18^, ^19^] Very recently, the PestE variant I208A L209F N288A was reported to catalyse the formation of two 4‐hydroxycinnamides in good to excellent yield^[25]^ and SpL has been reported for the *N*‐acylation of ethyl esters with aromatic and aliphatic amines in buffer, on preparative scales.^[20]^ The fact that PestE is capable of catalysing formation of amides from larger, more synthetically relevant acyl donors makes it a promising enzyme for industrial application.

We have also used PestE to form the amide bond in the leukemia drug tamibarotene, demonstrating its synthetic potential with more demanding pharmaceutical targets. In addition, a 3‐step enzymatic cascade for the formation of a benzylamide from waste plastic demonstrates the possibility for upcycling used PET to value added chemicals.

Several other amides have been formed from the corresponding carboxylic acids via methyltransferase FtpM and PestE, although in modest yields (8–16 %). An alternative cascade via hydrolysis of PET with PETase to MHET and subsequent amidation by PestE has limited efficiency at this stage, so future work should focus on improving both PETase and PestE activity to allow combination of hydrolysis and amidation steps. We have shown that protein engineering of PestE has been successful in improving the acyltransferase activity and rate of amide formation, including for the formation of tamibarotene (36 % conversion with F33L and F33A variants). This reaction was also achieved on a preparative scale and the tamibarotene product isolated, demonstrating the applicability of our system.

A current limitation with some substrates is the high amine excess needed to overcome the competing hydrolysis, so increasing amidation activity over hydrolysis is needed for larger scale application. Future work will therefore focus on further rounds of mutagenesis of PestE to generate a highly efficient acyl transferase and expand substrate scope to more complex, secondary cyclic or aliphatic amines/diamines. Our cascade also has a costly SAM (S‐adenosyl methionine) requirement for the methylation step, so future work will also involve investigation into SAM recycling methods or use of accessible esters such as MHET. Work is in progress to develop a whole‐cell system where FtpM and PestE can be co‐expressed to reduce economic burden of the cofactor requirement. This would negate the need to individually produce and purify each protein and avoid the requirement for exogenous cofactor addition.

## Conflict of Interests

The authors declare no conflict of interest.

1

## Supporting information

As a service to our authors and readers, this journal provides supporting information supplied by the authors. Such materials are peer reviewed and may be re‐organized for online delivery, but are not copy‐edited or typeset. Technical support issues arising from supporting information (other than missing files) should be addressed to the authors.

Supporting Information

## Data Availability

The data that support the findings of this study are available in the supplementary material of this article.
