# Supplementary material for: Development of the Esterase PestE for Amide Bond Synthesis Under Aqueous Conditions: Enzyme Cascades for Converting Waste PET into Tamibarotene
Source: Angew Chem Int Ed Engl. 2024 Nov 19;64(2):e202414162. doi: 10.1002/anie.202414162 (PMC11720379; doi:10.1002/anie.202414162)

Angewandte Chemie
Eine Zeitschrift der Gesellschaft Deutscher Chemiker

Supporting Information

Development of the Esterase PestE for Amide Bond Synthesis Under Aqueous Conditions: Enzyme Cascades for Converting Waste PET into Tamibarotene

*E. Goulding, L. C. Ward, F. E. Allan, D. Dittman, J. E. Salcedo-Sora, A. J. Carnell**

Supporting Information
©Wiley-VCH 2021
69451 Weinheim, Germany

Development of the Esterase PestE for Amide Bond Synthesis under Aqueous Conditions: Enzyme Cascades for Converting Waste PET to Tamibarotene.

Ellie Goulding, Lucy C. Ward, Faye E. Allan, Drew Dittman, Jesus E. Salcedo-Sora, and Andrew J. Carnell*

Abstract: A growing number of hydrolase enzymes show promiscuous acyl transferase activity, even under aqueous conditions. Here we report, for the first time, the ability of *Pyrobaculum calidifontis* VA1 esterase (PestE) to catalyse the formation of a wide range of amides in buffer, where the acyl donor forms a significant structural component in the amide product. The reactions occur under mild conditions and can achieve conversions up to 97% in 6 h for formation of *N*-benzylfuranamide as the model reaction. We demonstrate PestE's potential in enzyme cascades to make amides from waste PET plastic and the conversion of the terephthalic acid product to tamibarotene, a drug with activity against acute leukemia. Rational mutagenesis led to identification of PestE variants F33L_F289A and F33L. F33L_F289A increased conversion of *N*-benzylfuranamide by 1.2-fold, and F33L gave a 4-fold increase in conversion to tamibarotene.

DOI:

Table of Contents

1. Experimental Procedures 3
1.1 Enzymes 3
1.2 Protein expression 3
1.2.1 General methods for cell harvesting, lysis and purification 3
1.2.2 PestE and mutants 3
1.2.3 FtpM 3
1.2.4 LCC (WCCG) 3
1.2.5 PETase and TfH 4
1.2.6 SDS-PAGE analysis 4
1.3 Pure protein yields 5
Table S1- Purified protein yields for enzymes used in this study. 5
1.4 Enzyme activity analysis 6
1.4.1 Biotransformation analysis and sample preparation 6
1.4.2 PestE reaction conditions. 6
1.4.3 Tamibarotene formation reaction conditions 7
1.4.4 PET production and hydrolysis 7
1.4.5 PET to MMT cascade reaction conditions 7
1.4.6 Terephthalic acid to amide cascade reaction conditions 7
1.4.7 RP-HPLC retention times 7
1.5 Synthetic methods 17
1.5.1 General equipment and analytical methods 17
1.5.2 Synthesis of furanamide product standards 17
1.5.3 Biotranformation scale-ups 21
1.6 Computational methods 22
1.6.1 Ligand docking 22
2. Results and Discussion 22
2.1 Initial enzyme screen 22
2.2 Substrate ratio controls 23
2.3 Model reaction optimization- enzyme loading 23
2.4 Model reaction optimization- ester concentration 24
2.5 Model reaction optimization- pH 24
2.6 Model reaction optimization- temperature 25
2.7 Reaction conversions for PestE catalysed amide formation- substrates not accepted 25
2.8 PestE catalysed tamibarotene formation- reaction optimization. 26
2.9 PestE catalysed amide formation from MHET and benzylamine. 27
2.10 Terephthalic acid to amides cascade catalysed by FtpM and PestE. 27
2.11 Time course reactions for all PestE mutants. 28
2.12 Representative chromatograms 29
References 31
Author Contributions 31
NMR spectra 31

1. Experimental Procedures

1.1 Enzymes

CalA (NovoCor) and CalB (Lipozyme) were purchased from Strem chemicals inc. For PETase, TfH, PestE and MsAcT plasmids containing gene constructs were provided by GeneMill (MerseyBio, University of Liverpool). AddGene Is-PETase (DNA sequences in Appendix) and TfH (Uniprot accession number: Q47RJ6) were codon optimised and subcloned into a pET21a vector with a C-terminally coded histidine tag and a T7 inducible promoter. PestE (PBD code: 3ZWQ) and MsAcT (GenBank accession code: ABK70783, PBD code: 2Q0Q) were subcloned into a PET-based golden gate acceptor vector with a C-terminally coded histidine tag and a T7 inducible promoter. PestE mutant plasmids were purchased from TWIST Biosciences in a pET28a vector with a C-terminally coded histidine tag and a T7 inducible promoter.

1.2 Protein expression

1.2.1 General methods for cell harvesting, lysis and purification

After growth and protein expression, cells were harvested by centrifugation (4000g, 4°C 10 min) before resuspending in buffer A (50 mM NaPi pH 7.4, 300 mM NaCl) supplemented with DNase (2.5 μg/mL) and protease inhibitor for lysis. Cells were lysed by ultrasonication on ice (10s on, 30s off, 2 min 40s, 4 cycles, 50% energy, 30% resistance). Cell debris was removed by centrifugation (15, 000 g, 4°C, 60 min). The supernatant was collected and filtered using a 0.45 μm syringe filter prior to loading onto either a 1 mL or 5 mL nickel Histrap Fast Flow affinity purification column. The column was washed with buffer A containing 50 mM imidazole and eluted with an increasing gradient of imidazole (to 500 mM) at a flow rate of 5 mL/min, using an ÄKTA Start protein purification system. Fractions were analysed by SDS-PAGE and purified protein buffer exchanged using a PD-10 Sephadex column into 100 mM NaPi buffer pH 8.0. When necessary, protein samples were concentrated by centrifugation in a 10 kDa centrifugal filter unit (4000g, 4°C) and purified protein was stored at -80°C with the addition of 10% glycerol.

1.2.2 PestE and mutants

PestE and mutants were transformed into *E. coli* BL21 (DE3) cells. For expression, a single colony of cells was picked from overnight LB agar plates to inoculate LB media (10 mL) containing kanamycin (50 μg/mL) at 37°C, 120 rpm, for 16 h. This was used to inoculate LB (1 L containing kanamycin, 50 μg/mL), prior to incubation at 37°C, 180 rpm until the culture reached an OD600 of 0.6-0.8. Protein expression was induced by the addition of IPTG (1 mM) and temperature reduced to 18°C for further incubation (16 h). Cell harvesting, lysis and protein purification were performed as in 1.2.1.

1.2.3 FtpM

As described by Ward *et al.*[1] FtpM was transformed into *E. coli* SoluBL21 (DE3) cells (Genlantis). For expression, a single colony of cells was picked from overnight LB agar plates to inoculate LB media (10 mL) containing ampicillin (100 μg/mL) at 37°C, 120 rpm, for 16 h. This was used to inoculate LB (1 L containing ampicillin, 100 μg/mL), prior to incubation at 37°C, 180 rpm until the culture reached an OD600 of 0.6-0.8. Protein expression was induced by the addition of IPTG (1 mM) and temperature reduced to 16°C for further incubation (16 h). Cell harvesting, lysis and protein purification were performed as in 1.2.1. Samples were buffer exchanged into 50 mM MES buffer pH 6.0.

1.2.4 LCC (WCCG)

As described by Sadler *et al*.[2] LCC (WCCG) was transformed into *E. coli* BL21 (DE3) cells (Genlantis). For expression, a single colony of cells was picked from overnight LB agar plates to inoculate LB media (10 mL) containing ampicillin (100 μg/mL) at 37°C, 120 rpm, for 16 h. This was used tinoculate auto-induction TB (1 L containing ampicillin, 100 μg/mL), prior to incubation at 22°C, 180 rpm for 24 h. Cell harvesting, lysis and protein purification were performed as in 1.2.1. Samples were buffer exchanged into 20 mM NaPi pH 7.4, 300 mM NaCl.

1.2.5 PETase and TfH

As described by Parisi *et al.*[3] PETase and TfH were transformed into *E. coli* SoluBL21 (DE3) cells. For expression, a single colony of cells was picked from overnight LB agar plates to inoculate LB media (10 mL) containing ampicillin (100 μg/mL) at 37°C, 120 rpm, for 16 h. This was used to inoculate auto-induction TB (1 L containing ampicillin, 100 μg/mL), prior to incubation at 37°C, 180 rpm until the culture reached an OD600 of 0.6-0.8, when temperature was reduced to 18°C for further incubation (16 h). Cell harvesting, lysis and protein purification were performed as in 1.2.1.

1.2.6 SDS-PAGE analysis

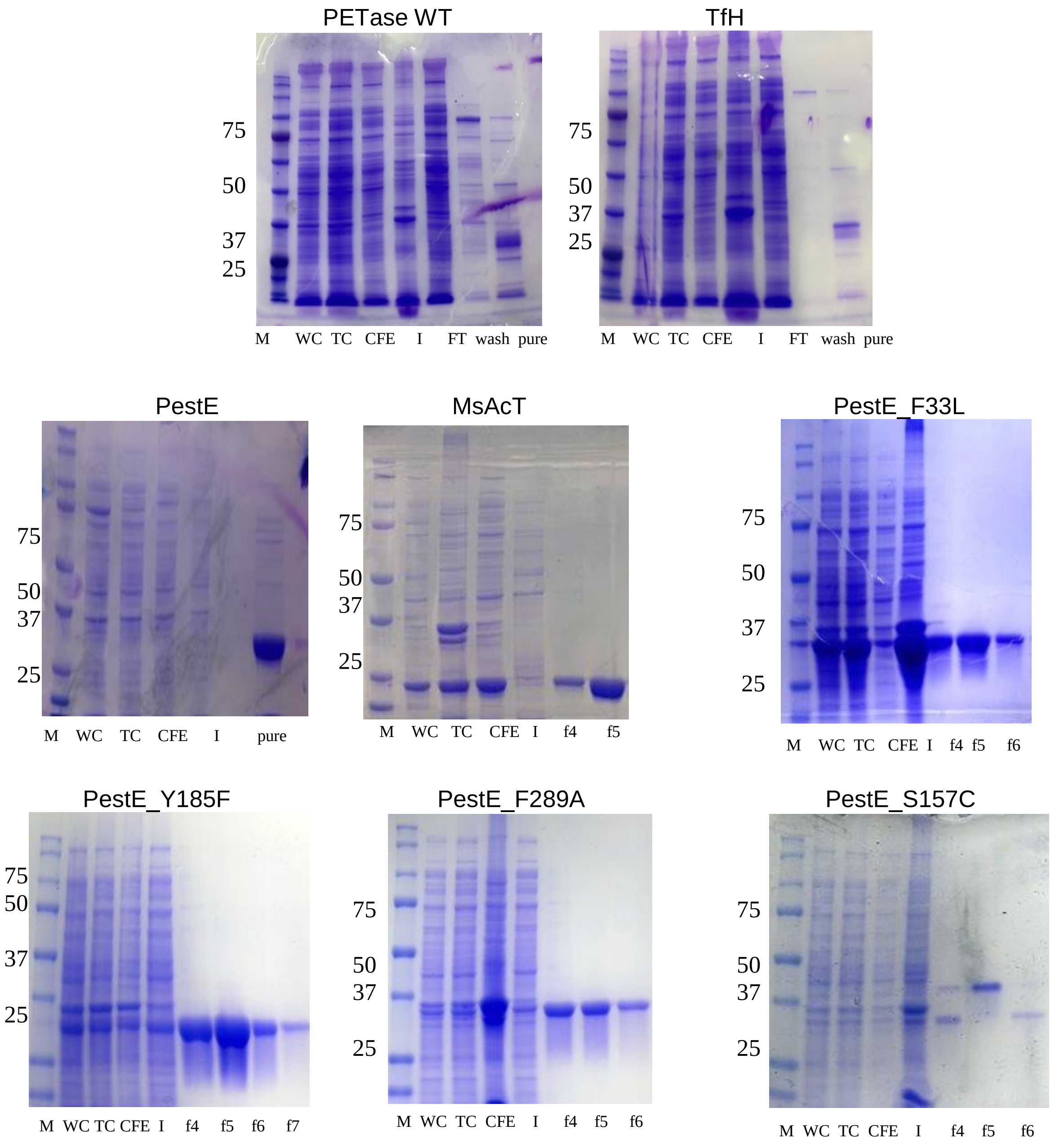

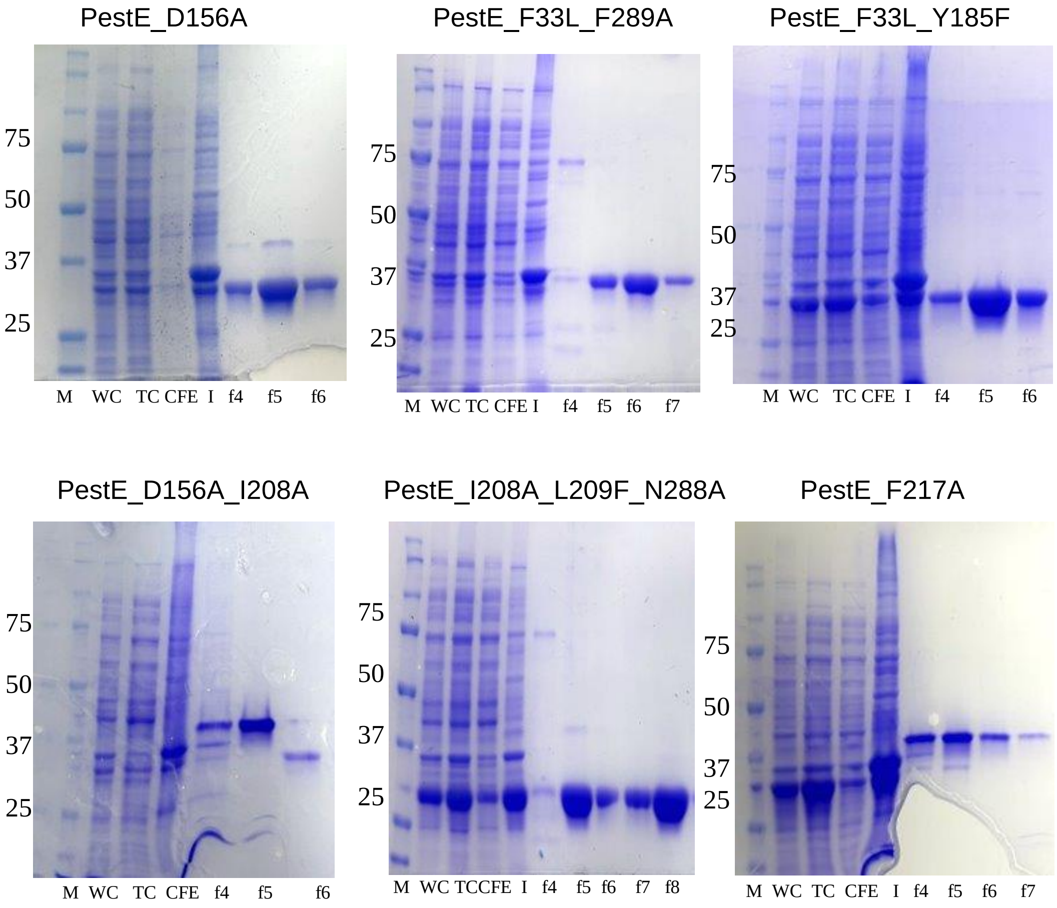

Figure S1- SDS-PAGE analysis for PETase, TfH, MsAcT, PestE and mutants. M= lane marker, WC= whole cell content, TC= total cell content, CFE= cell-free extract, I= insoluble fraction, FT= flow through, wash= 10% buffer B wash.

1.3 Pure protein yields

Table S1- Purified protein yields for enzymes used in this study.

Mutant	Protein yield (mg/L)
PETase	5
TfH	8
PestE	20

MsAcT	16
FtpM	60
PestE_F33L	14
PestE_D156A	16
PestE_S157C	7
PestE_Y185F	36
PestE_F217A	44
PestE_F289A	34
PestE_F33A	20
PestE_F33L_F289A	22
PestE_F33L_Y185F	34
PestE_D156A_I208A	17
PestE_I208A_L209F_N288A	56

1.4 Enzyme activity analysis

1.4.1 Biotransformation analysis and sample preparation

All reverse phase HPLC was performed on an Agilent 1260 Infinity system equipped with a G1311C quaternary pump, G1329B autosampler unit, G1316A temperature-controlled column compartment and a G13150 diode array detector (DAD). A ZORBAX Eclipse XDB-C18 analytical column (Agilent) was used with dimensions of 150 mm length, 4.6 mm diameter, 5 μm particle size. LCMS analysis was performed at University of Manchester on an Agilent 1390 Infinity II UPLC system equipped with an InfinityLab LC/MSD XT and G6011B Quiet Cover MS. Samples were run using a gradient method of buffer A (H_2O, 0.1% formic acid) to buffer B (MeCN, 0.1% formic acid), 0.8 mL/min, 8 min, 254 or 240 nm. Unless otherwise stated, biotransformations were quenched with 2% trifluoroacetic acid (TFA) and diluted x5 in MeOH prior to centrifugation using a benchtop centrifuge (13, 000 rpm, 3 min) to remove protein precipitate. 100 μL of the sample was then transferred to a glass microvolume insert (Agilent) in a 2 mL screw top HPLC vial (Agilent) prior to RP-HPLC analysis as in section 5.1.4. Unless otherwise stated, an injection volume of 10 μL was used and chromatograms were monitored at 254 nm or 240 nm. Samples were run using a gradient method of buffer A (95% H_2O, 5% MeOH, 0.1% TFA) to buffer B (90% MeOH, 10% H_2O, 0.1% TFA), 0.6 mL/min, 35 min, 254 or 240nm. Chromatograms from biotransformations were compared to retention times of authentic standards ran under the same conditions for product identification. Final conversions of substrate to products were quantified by adjusting peak areas with the response factors from a 1:1:1 standard of substrate: product(s). Where authentic product standards could not be attained, amide product peaks were identified by LC-MS, and conversions quantified by a calibration curve of ester concentration and acid concentration to monitor substrate depletion and acid formation.

1.4.2 PestE reaction conditions.

For analytical-scale biotransformations, unless otherwise stated, ester substrate (10 mM), amine hydrochloride (100 mM) and enzyme (100 μM) in 300 mM Tris buffer (pH 9.0) was added to a 1.5 mL Eppendorf tube, with a final volume of 100 μL. Reactions were incubated at 25°C with shaking (250 rpm) for 16 h. Control reactions were also set up with enzyme storage buffer replacing the enzyme. Reactions were quenched and analysed by RP-HPLC as in 1.4.1.

1.4.3 Tamibarotene formation reaction conditions

For analytical-scale biotransformations, unless otherwise stated, monomethyl terephthalate substrate (5 mM), amine (10 mM) and PestE (100 μM) in 100 mM NaPi buffer (pH 8.0) was added to a 1.5 mL Eppendorf tube, with a final volume of 100 μL. Reactions were vigorously vortexed prior to incubation at 25°C with shaking (250 rpm) for 16 h. Control reactions were also set up with enzyme storage buffer replacing the enzyme. Reactions were quenched and analysed by RP-HPLC as in 1.4.1.

1.4.4 PET production and hydrolysis

A 500 mL plastic Pepsi Max bottle (20 g) was washed and cut into approximately 5 mm x 5 mm pieces (9.1 g). Finely ground PET (8.4 g) was acquired by covering the plastic in liquid nitrogen and using an A11 basic analytical mill equipped with an 80 mL milling chamber (IKA Mills). Reactions were assembled to contain a final concentration of 10 µM LCC (WCCG) with PET (20 mg) in 100 mM Tris buffer pH 9 alongside non-enzyme controls. Reaction mixtures were incubated for 72 hours at 72°C with shaking at 400 rpm. After incubation, an equivalent volume of 10% TFA was added and reaction mixtures were centrifuged (13,000 rpm, 3 minutes) on a table-top centrifuge ahead of HPLC analysis as in 1.4.1. Terephthalic acid was quantified by calibration curve with 1 mM caffeine as internal standard.

1.4.5 PET to MMT cascade reaction conditions

Unless otherwise stated, reactions were set up to include LCC (WCCG) (20 μM), PET (5 mg) in Tris buffer (pH 9.0) in a 2 mL screw top vial and incubated at 400 rpm, 72°C. After 48 h, LCC (WCCG) was heat inactivated at 100°C for 3 min, prior to addition of methylation reaction components: FtpM (40 μM), SAM (4 mM), SAH-nuc (8 μM). Reactions were then incubated for a further 2.5 h at 25°C, 250 rpm. When required, acylation reaction components were added at this point: PestE (100 μM) and amine (50 mM) and further incubated for another 18 h at 25°C, 250 rpm. Reactions were quenched and analysed by RP-HPLC as in 1.4.1. Reaction conversions for the first step was monitored by taking 100 μL sample and quenching with 2% TFA and diluting in MeOH x2 prior to RP-HPLC analysis as in section 1.4.1- TA was quantified by comparison to a calibration curve of terephthalic acid. Control reactions were also set up with enzyme storage buffer replacing the enzyme.

1.4.6 Terephthalic acid to amide cascade reaction conditions

For analytical-scale biotransformations, unless otherwise stated, the following reaction components were added to a 1.5 mL Eppendorf tube: FtpM (200 μM), terephthalic acid (5 mM), SAM (50 mM), SAH-nuc (8 μM) in 100 mM NaPi buffer (pH 8.0), with a final volume of 100 μL. Reactions were incubated at 25°C with shaking (250 rpm). After 16 h acylation reaction components were added to the reaction mixture: PestE (100 μM) and amine (50 mM, except for tamibarotene reaction, where the amine **95** was added at 10 mM), and left incubating for a further 16 h. Control reactions were also set up with enzyme storage buffer replacing the enzyme. Reactions were quenched and analysed by RP-HPLC as in 1.4.1.

1.4.7 RP-HPLC retention times

Table S2-RP-HPLC retention times. RP-HPLC conditions described in Chapter 5. (a)= retention time observed at 254 nm. (b)= retention time observed at 240 nm. *= product identity determined by LCMS.

Entry	Time (min)
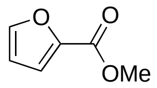 **1**	16.3 (a)
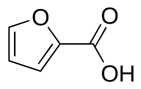 **4**	10.4 (a)

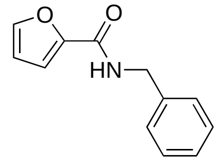

3

20.3 (a)

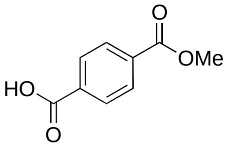

54

20.4 (b)

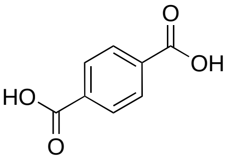

71

16.7 (b)

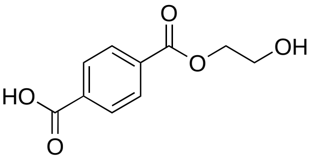

91

18.5 (b)

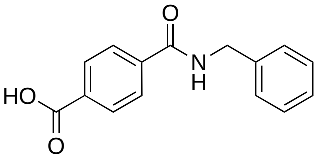

88

21.6 (b)

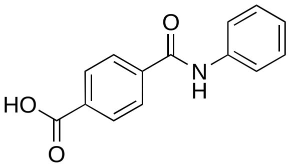

94

21.3 (b)*

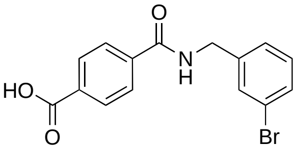

95

23.3 (b)*

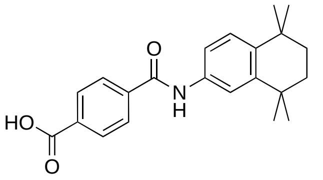

28.6 (b)

93

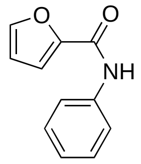

21.2 (a)

32

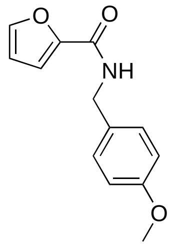

20.4 (a)

22

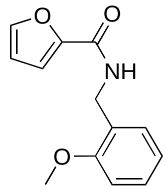

21.0 (a)

23

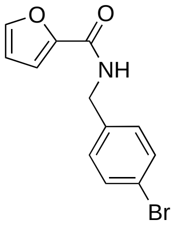

23.1 (a)

24

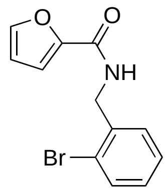

22.6 (a)

25

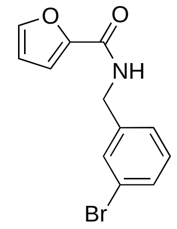

22.9 (a)

26

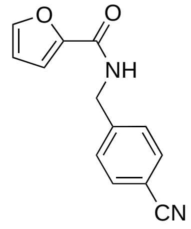

18.9 (a)

27

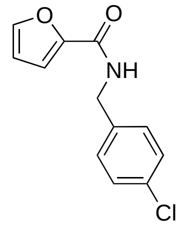

22.6 (a)

28

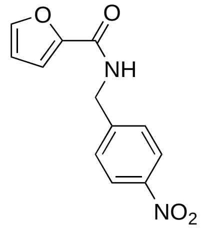

19.5 (a)*

29

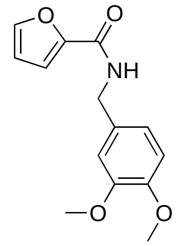

19.2 (a)

30

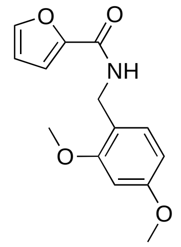

21.8 (a)

31

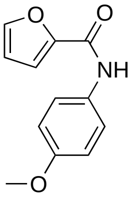

20.2 (a)

33

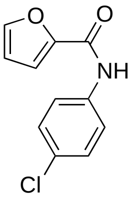

23.1 (a)

34

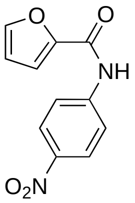

19.5 (a)*

35

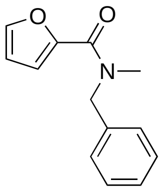

22.4 (a)

36

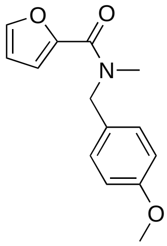

23.2 (a)

37

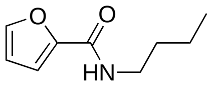

19.5 (a)

38

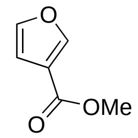

17.2 (a)

39

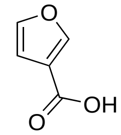

11.6 (a)

56

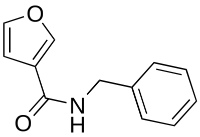

19.9 (a)*

73

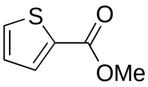

21.1 (a)

40

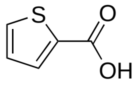

20.2 (a)

57

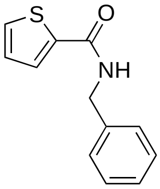

21.4 (a)

74

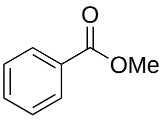

23.0 (b)

41

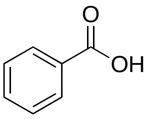

20.6 (b)

58

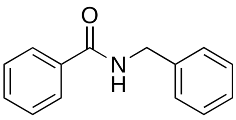

23.6 (b)

75

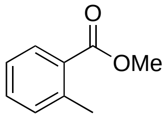

20.2 (b)

42

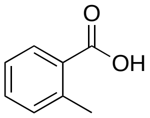

18.9 (b)

59

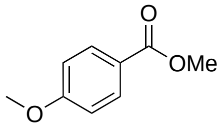

22.2 (b)*

43

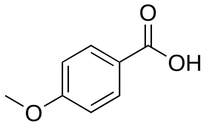

19.6 (b)

60

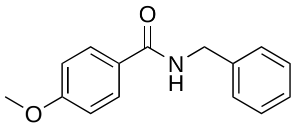

22.4 (b)*

77

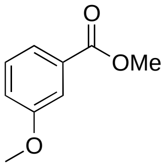

22.5 (b)

44

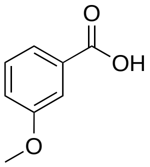

20.0 (b)

61

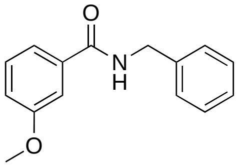

23.0 (b)*

78

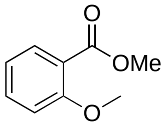

20.3 (b)

45

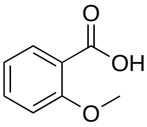

17.2 (b)

62

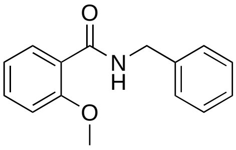

23.0 (b)*

79

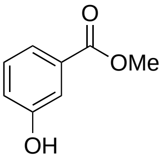

19.0 (b)

46

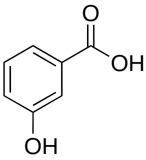

15. 0 (b)

63

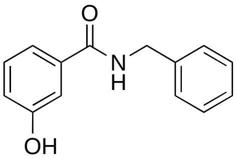

20.1 (b)*

80

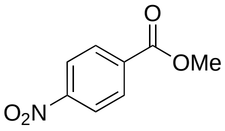

21.7 (b)

47

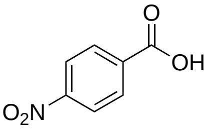

19.0 (b)

64

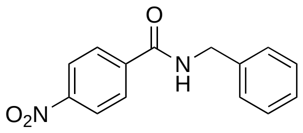

22.3 (b)*

81

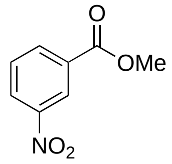

21.6 (b)

48

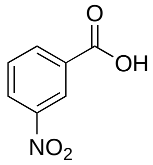

19.2 (b)

65

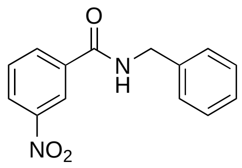

22.7 (b)*

82

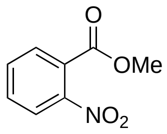

19.0 (b)

49

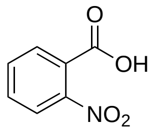

14.1 (b)

66

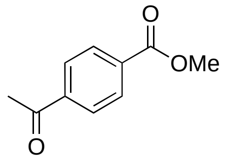

20.7 (b)

50

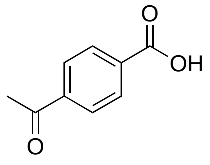

18.1 (b)

67

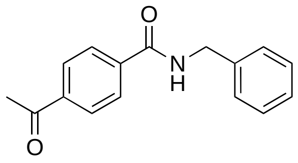

21.4 (b)*

84

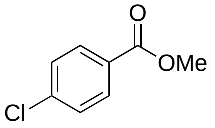

24.5 (b)

51

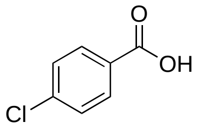

22.7 (b)

68

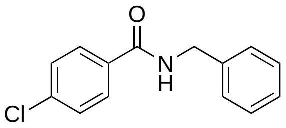

24.8 (b)*

85

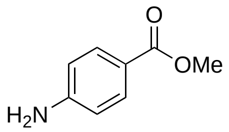

13.3 (b)

52

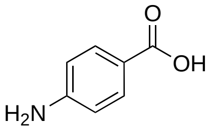

4.7 (b)

69

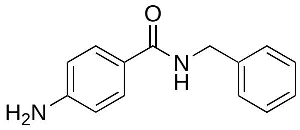

16.8 (b)*

86

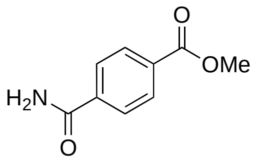

16.5 (b)

53

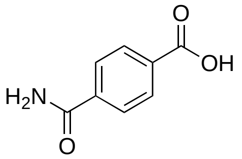 **70**	11.7 (b)
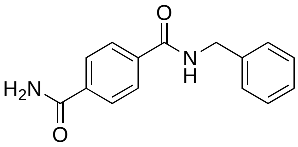 **87**	18.9 (b)*
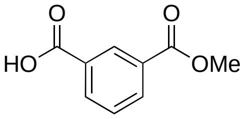 **55**	19.9 (b)
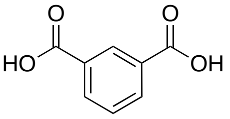 **72**	17.2 (b)

1.5 Synthetic methods

1.5.1 General equipment and analytical methods

Commercial reagents and solvents were purchased from Sigma Aldrich, TCI Chemicals, Fluorochem or BLD Pharm and used without further purification. Thin layer chromatography was performed using UV254 sensitive, silica gel coated, aluminium TLC plates purchased from Merck. Visualisation was achieved by UV fluorescence or basic $KMnO_4$ solution. NMR spectra were recorded using a Bruker 400 MHz spectrometer in the deuterated solvent reported. Chemical shifts (δ) are reported in ppm and coupling constants (J) are quoted in Hz to the nearest 0.5 Hz. Signal multiplicities are assigned as singlets (s), doublets (d), triplets (t), quartets (q), multiplets (m) or broad (br). Mass spectra were provided by the University of Liverpool Analytical Services Department.

1.5.2 Synthesis of furanamide product standards

Furoyl chloride (2.4 mmol, 1.2 equiv) was added to dichloromethane (DCM, 30 mL) in a 100 mL round-bottomed flask under a nitrogen atmosphere. Anhydrous triethylamine (4 mL) was added, prior to amine addition (2 mmol, 1 equiv) at 0°C. The mixture was left stirring on ice for 2 h, followed by stirring at room temperature overnight under a nitrogen atmosphere. Reaction progress was monitored by TLC (hexane:ethyl acetate, 70:30). After 18 h, the reaction was extracted with 1 M HCl (30 mL x 2) and washed with sodium bicarbonate (30 mL), H_2O (30 mL) and brine (30 mL). The organic layer was then dried with $MgSO_4$, filtered under vacuum and the solvent removed *in vacuo*.

***N*-benzylfuran-2-carboxamide**

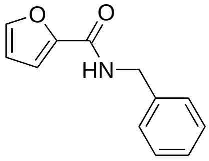

3

^{1}H NMR (400 MHz, MeOD) δ 7.65 – 7.62 (m, 1H), 7.36 – 7.27 (m, 4H), 7.26 – 7.20 (m, 1H), 7.15 – 7.11 (m, 1H), 6.59 – 6.55 (m, 1H), 4.54 (s, 2H). ^{13}C NMR (101 MHz, MeOD) δ 159.41, 147.67, 144.91, 138.62, 128.13, 127.13, 126.82, 113.95, 111.55, 42.31. Structure consistent with literature report.[4]

_N_-phenylfuran-2-carboxamide

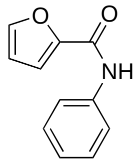

32

^{1}H NMR (400 MHz, MeOD) δ 7.72 – 7.66 (m, 3H), 7.33 (t, *J* = 7.9 Hz, 2H), 7.25 (d, *J* = 3.4 Hz, 1H), 7.13 (t, *J* = 7.4 Hz, 1H), 6.65 – 6.59 (m, 1H). ^{13}C NMR (101 MHz, MeOD) δ 157.49, 147.61, 145.25, 137.73, 128.42, 124.30, 120.81, 114.75, 111.83.

_N_-benzylthiopehene-2-carboxamide

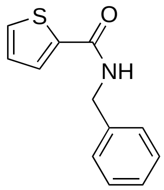

74

^{1}H NMR (400 MHz, MeOD) δ 7.71 (dd, *J* = 3.6, 0.6 Hz, 1H), 7.64 – 7.62 (m, 1H), 7.38 – 7.28 (m, 4H), 7.20-7.26 (m, *J* = 8.9, 4.3 Hz, 1H), 7.09-7.14 (m, *J* = 4.8, 3.9 Hz, 1H), 4.53 (s, 2H). ^{13}C NMR (101 MHz, MeOD) δ 162.98, 138.80, 138.72, 130.30, 128.24, 128.13, 127.40, 127.12, 126.80, 42.97. Structure consistent with literature report.[4]

_N_-(3,4-dimethoxybenzyl) furan-2-carboxamide

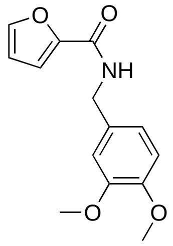

30

^{1}H NMR (400 MHz, MeOD) δ 7.65 – 7.61 (m, 1H), 7.13 (d, *J* = 3.2 Hz, 1H), 6.97 (s, 1H), 6.90 – 6.85 (m, 2H), 6.58 – 6.55 (m, 1H), 3.80 (s, 3H), 3.78 (s, 3H). ^{13}C NMR (101 MHz, MeOD) δ 159.2, 149.09, 148.37, 147.64, 144.90, 131.48, 119.85, 113.92, 111.56, 111.41, 55.09, 55.00, 42.13.

_N_-(4-chlorobenzyl) furan-2-carboxamide

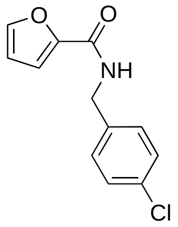

28

^{1}H NMR (400 MHz, MeOD) δ 7.68 – 7.64 (m, 1H), 7.32 (s, 4H), 7.14 (d, *J* = 3.1 Hz, 1H), 6.62 – 6.55 (m, 1H), 4.52 (s, 2H). ^{13}C NMR (101 MHz, MeOD) δ 159.42, 147.52, 144.97, 137.53, 132.56, 128.75, 128.16, 114.05, 111.57, 41.62. [M+H]+: 236.0478. Found [M+H]+: 236.0475.

_N_-(2,4-dimethoxybenzyl) furan-2-carboxamide

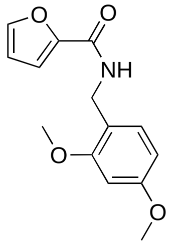

31

^{1}H NMR (400 MHz, MeOD) δ 7.64 – 7.62 (m, 1H), 7.16 (d, *J* = 8.3 Hz, 1H), 7.13 – 7.09 (m, 1H), 6.58 – 6.55 (m, 1H), 6.55 – 6.52 (m, 1H), 6.47 (dd, *J* = 8.3, 2.2 Hz, 1H), 4.47 (s, 2H), 3.84 (s, 3H), 3.77 (s, 3H). ^{13}C NMR (101 MHz, MeOD) δ 160.62, 159.23, 158.34, 147.69, 144.79, 129.08, 118.22, 113.75, 111.50, 103.87, 97.92, 54.49, 54.38, 37.55. [M+H]+: 262.1079. Found [M+H]+: 262.0977.

N-(4-chlorophenyl) furan-2-carboxamide

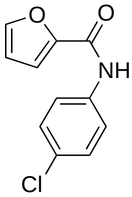

34

^{1}H NMR (400 MHz, MeOD) δ 7.79 – 7.60 (m, 3H), 7.29-7.35 (m, *J* = 8.8 Hz, 2H), 7.27 (d, *J* = 3.4 Hz, 1H), 6.71 – 6.59 (m, 1H). ^{13}C NMR (101 MHz, MeOD) δ 157.40, 147.42, 145.38, 136.67, 129.11, 128.36, 122.01, 114.96, 111.86. [M+H]+: 222.0322. Found [M+H]+: 222.0299.

N-(4-bromobenzyl) furan-2-carboxamide

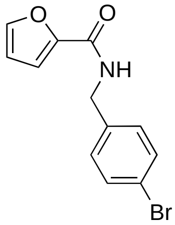

24

^{1}H NMR (400 MHz, MeOD) δ 7.64 (d, *J* = 0.7 Hz, 1H), 7.45 (d, *J* = 8.3 Hz, 2H), 7.24 (d, *J* = 8.3 Hz, 2H), 7.12 (d, *J* = 3.1 Hz, 1H), 6.56 (dd, *J* = 3.3, 1.7 Hz, 1H), 4.48 (s, 2H). ^{13}C NMR (101 MHz, MeOD) δ 159.43, 147.50, 144.98, 138.01, 131.19, 129.08, 120.49, 114.07, 111.59, 41.69. [M+H]+: 279.9973. Found [M+H]+: 279.9968.

N-(4-methoxybenzyl) furan-2-carboxamide

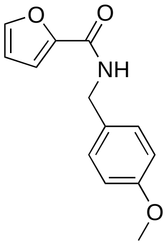

22

^{1}H NMR (400 MHz, MeOD) δ 7.71-7.75 (m, *J* = 0.9 Hz, 1H), 7.35 (d, *J* = 8.6 Hz, 2H), 7.21 (s, 1H), 6.96 (d, *J* = 8.7 Hz, 2H), 6.66 (dd, *J* = 3.4, 1.7 Hz, 1H), 4.56 (s, 2H), 3.85 (s, 3H). ^{13}C NMR (101 MHz, MeOD) δ 159.29, 159.01, 147.65, 144.86, 130.62, 128.52, 114.12, 113.50, 111.52, 54.28, 41.79.

N-benzyl-N-methylfuran-2-carboxamide

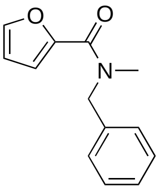

36

^{1}H NMR (400 MHz, MeOD) δ 7.68 (d, *J* = 10.8 Hz, 1H), 7.43 – 7.21 (m, 5H), 7.11 (s, 1H), 6.58 (s, 1H), 4.74 (s, 2H), 3.23 (s, 3H).

_N_-(2-bromobenzyl) furan-2-carboxamide

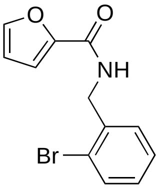

25

^{1}H NMR (400 MHz, MeOD) δ 7.67 (d, *J* = 0.8 Hz, 1H), 7.57 (d, *J* = 7.9 Hz, 1H), 7.40 – 7.26 (m, 2H), 7.13-7.19 (m, *J* = 5.9 Hz, 2H), 6.59 (dd, *J* = 3.4, 1.7 Hz, 1H), 4.61 (s, 2H). ^{13}C NMR (101 MHz, MeOD) δ 159.50, 147.44, 145.07, 137.10, 132.41, 128.60, 128.44, 127.33, 122.54, 114.20, 111.63, 42.78. [M+H]+: 279.9973. Found [M+H]+: 279.9966.

_N_-(2-methoxybenzyl) furan-2-carboxamide

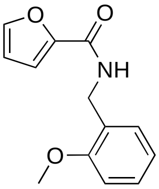

23

^{1}H NMR (400 MHz, MeOD) δ 7.65 (d, *J* = 0.7 Hz, 1H), 7.22-7.28 (m, *J* = 7.5 Hz, 2H), 7.13 (d, *J* = 3.4 Hz, 1H), 6.97 (d, *J* = 8.1 Hz, 1H), 6.90 (t, 1H), 6.58 (dd, *J* = 3.4, 1.7 Hz, 1H), 4.55 (s, 2H), 3.87 (s, 3H). ^{13}C NMR (101 MHz, MeOD) δ 159.38, 157.30, 147.65, 144.86, 128.24, 127.94, 125.94, 120.02, 113.83, 111.53, 110.01, 54.46, 37.81. [M+H]+: 218.0639. Found [M+H]+: 218.0635.

_N_-(4-methoxyphenyl) furan-2-carboxamide

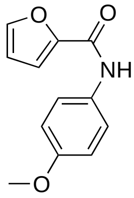

33

^{1}H NMR (400 MHz, MeOD) δ 7.71 (s, 1H), 7.57 (d, *J* = 9.0 Hz, 2H), 7.23 (d, *J* = 3.3 Hz, 1H), 6.91 (d, *J* = 9.0 Hz, 2H), 6.62 (dd, *J* = 3.3, 1.6 Hz, 1H), 3.79 (s, 3H). ^{13}C NMR (101 MHz, MeOD) δ 157.41, 156.89, 147.70, 145.12, 130.55, 122.59, 114.44, 113.57, 111.76, 54.47. [M+H]+: 218.0817. Found [M+H]+: 218.0763.

_N_-(4-cyanobenzyl) furan-2-carboxmaide

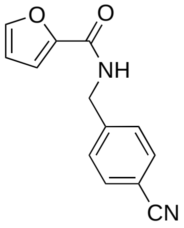

27

^{1}H NMR (400 MHz, MeOD) δ 7.68-7.73 (m, *J* = 6.1, 4.6 Hz, 3H), 7.53 (d, *J* = 8.3 Hz, 2H), 7.15 (d, *J* = 3.4 Hz, 1H), 6.61 (dd, *J* = 3.4, 1.7 Hz, 1H), 4.62 (s, 2H). ^{13}C NMR (101 MHz, MeOD) δ 159.54, 147.39, 145.10, 144.63, 132.03, 127.90, 118.27, 114.22, 111.63, 110.54, 41.94. [M+H]+: 227.0821. Found [M+H]+: 227.0802.

_N_-(3-bromobenzyl) furan-2-carboxamide

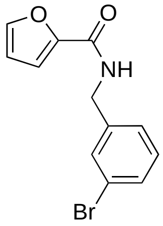

26

^{1}H NMR (400 MHz, MeOD) δ 7.64-7.67 (m, *J* = 0.9 Hz, 1H), 7.51 (s, 1H), 7.40 (d, *J* = 7.9 Hz, 1H), 7.31 (d, *J* = 7.7 Hz, 1H), 7.20-7.26 (m, *J* = 7.8 Hz, 1H), 7.14 (d, *J* = 3.5 Hz, 1H), 6.58 (dd, *J* = 3.4, 1.7 Hz, 1H), 4.51 (s, 2H). ^{13}C NMR (101 MHz, MeOD) δ 159.45, 147.45, 146.41, 145.03, 141.36, 130.14, 129.87, 125.94, 122.03, 114.14, 111.60, 41.71. $C_{12}H_{11}BrNO_2$ [M+H]+: 279.9973. Found [M+H]+: 279.9966.

_N_-(4-methoxybenzyl)-_N_-methylfuran-2-carboxamide

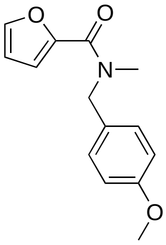

37

^{1}H NMR (400 MHz, MeOD) δ 7.84 (s, 3H), 7.24 – 7.10 (m, 2H), 7.04 (s, 1H), 6.99 – 6.84 (m, 2H), 6.62 (dd, *J* = 3.4, 1.8 Hz, 1H), 4.61 (s, 2H), 3.74 (s, 3H). ^{13}C NMR (101 MHz, MeOD) δ 159.01, 147.69, 145.32, 129.58, 128.44, 114.99, 114.49, 114.29, 111.82, 55.52, 40.46. [M+H]+: 246.1130. Found [M+H]+: 246.0975.

_N_-butylfuran-2-carboxamide

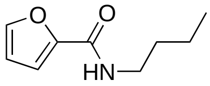

38

^{1}H NMR (400 MHz, MeOD) δ 7.42 (s, 1H), 7.10 (s, 1H), 6.56 – 6.36 (m, 1H), 3.39-3.46 (m, *J* = 20.1, 13.6, 6.8 Hz, 2H), 1.55-1.64 (m, *J* = 14.8, 7.3 Hz, 2H), 1.35-1.44 (m, *J* = 14.5, 7.3 Hz, 2H), 0.95 (t, *J* = 7.3 Hz, 3H). ^{13}C NMR (101 MHz, MeOD) δ 158.48, 148.13, 143.69, 113.94, 112.06, 38.87, 31.71, 20.04, 13.70. Structure consistent with literature report.[5]

1.5.3 Biotranformation scale-ups

PestE and PestE F33L_F289A catalysed formation of _N_-benzylfuranamide 3

Methyl 2-furoate (25 mM, from 250 mM stock in DMSO) and benzylamine hydrochloride (50 mM, from 1 M stock in H_2O) were added to a solution of 200 mM Tris buffer (pH 9.0) in a 50 mL round-bottomed flask. PestE (100 µM WT or F33L_F289A) was added to achieve a final reaction volume of 10 mL and the mixture was left stirring at room temperature for 24 h. The reaction was subsequently quenched with 12 M HCl to reach pH 2.0, and the amide product extracted with ethyl acetate (3 x 10 mL) prior to centrifugation (5 min, 4000 g, 4°C) to aid separation. Organic layers were then washed with 1 M HCl (30 mL), sodium bicarbonate (30 mL), H_2O (30 mL) and brine (30 mL). The organic layer was then dried with $NaSO_4$, filtered by gravity and the solvent removed *in vacuo* to yield the amide product as a white solid (WT: 31 mg, 62%, F33L_F289A: 36 mg, 72%).

Wild Type PestE:
^{1}H NMR (500 MHz, $CDCl_3$) δ 7.43-7.39 (m, 1H), 7.37 – 7.33 (m, 4H), 7.32 – 7.27 (m, 1H), 7.15 (d, J = 3.5 Hz, 1H), 6.67 (br s, 1H), 6.50 (dd, J = 3.5, 1.7 Hz, 1H), 4.61 (d, J = 5.9 Hz, 2H). ^{13}C NMR (126 MHz, $CDCl_3$) δ 158.38, 148.03, 144.01, 138.14, 128.89, 128.04, 127.76, 114.53, 112.31, 43.29. HRMS ESI [M+H]+: 202.0868. Found [M+H]+: 202.0866. Structure is consistent with literature report.[4]

PestE F33L_F289A:
^{1}H NMR (500 MHz, $CDCl_3$) δ 7.45 – 7.41 (m, 1H), 7.37 – 7.33 (m, 4H), 7.32 – 7.27 (m, 1H), 7.15 (d, J = 3.5 Hz, 1H), 6.66 (br s, 1H), 6.50 (dd, J = 3.6, 1.8 Hz, 1H), 4.61 (d, J = 5.8 Hz, 2H). ^{13}C NMR (126 MHz, $CDCl_3$) δ 158.38, 148.03, 144.01, 138.14, 128.89, 128.04, 127.76, 114.53, 112.31, 43.29. HRMS ESI [M+H]+: 202.0868. Found [M+H]+: 202.0866. Structure is consistent with literature report.[4]

PestE catalysed formation of tamibarotene 93

Monomethyl terephthalate (25 mM, from 1 M stock in DMSO) and amine **92** (50 mM, from 250 mM stock in DMSO) were added to a solution of 200 mM NaPi buffer (pH 8.0) in a 50 mL round-bottomed flask to a reaction volume of 15 mL. Vigorous vortexing was performed to solubilise the substrates, prior to the addition of PestE_F33A (50 µM) and the mixture was left stirring at room temperature for 24 h. The reaction was subsequently quenched with 12 M HCl to reach pH 2.0, and the amide product extracted with ethyl acetate (3 x 15 mL) prior to centrifugation (5 min, 4000 g, 4°C) to aid separation. Organic layers were then washed with 1 M HCl (2 x 45 mL), H_2O (45 mL) and brine (45 mL). The organic layer was then dried with $NaSO_4$, filtered by gravity and the solvent removed *in vacuo* to obtain the crude product as an orange solid. Purification by flash column chromatography on silica gel using DCM/MeOH/CH_3COOH (98/2/0.1) afforded the amide product as an off- white solid (19 mg, 15%).

^{1}H NMR (400 MHz, MeOD) δ 8.14 (d, *J* = 8.2 Hz, 2H), 8.00 (d, *J* = 8.1 Hz, 2H), 7.66 (d, *J* = 2.3 Hz, 1H), 7.46 (dd, *J* =8.6 and 2.3 Hz, 1H), 7.32 (d, *J* = 8.6 Hz, 1H), 1.74 – 1.70 (m, 4H), 1.31 (s, 6H), 1.29 (s, 6H). ^{13}C NMR (101 MHz, MeOD) δ 167.60, 166.48, 145.16, 141.24, 138.99, 135.67, 133.50, 129.45, 127.26, 126.54, 118.89, 118.69, 34.82, 33.97, 33.54, 30.84. HRMS (ESI) Calculated for $C_{22}H_{26}NO_3$, *m/z* $[M+H]^+$ requires 352.1913; found 352.1914. Structure is consistent with literature report.[6]

1.6 Computational methods

1.6.1 Ligand docking

The crystal structure of PestE was obtained using the PBD code: 3ZWQ. Acylated serine was constructed using the 'Build' functionality in PyMOL. Small molecule docking of the acylated PestE structure was performed using Webina 1.05, a web implementation of the AutoDock Vina algorithm.[7] Box centre coordinates were set to the catalytic serine and co-ordinates obtained through PyMOL, and box size was set to 15 Å beyond the ligand in each dimension. Autodock produced a number of ranked poses and predicted affinities which were examined in PyMOL. The top ranked pose was the only pose positioning the amine substrate with the amine nitrogen in the correct orientation for nucleophilic attack, so was used for subsequent analysis.

2. Results and Discussion

2.1 Initial enzyme screen

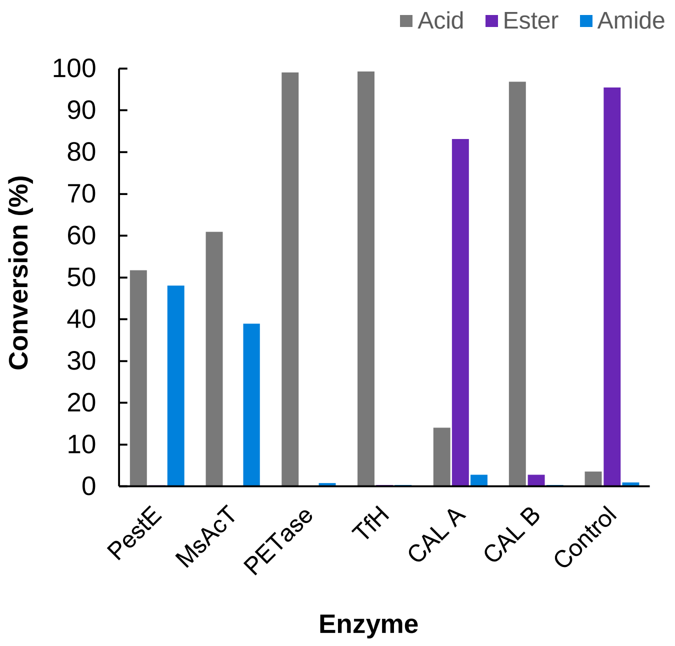

Figure S2- Enzymatic *N*-benzylfuranamide formation from methyl furoate and benzylamine HCl. Conditions: methyl furoate (10mM), benzylamine HCl (10mM), enzyme (50 µM), 200mM Tris buffer pH 9, 25°C, 200 rpm, 18 h.

2.2 Substrate ratio controls

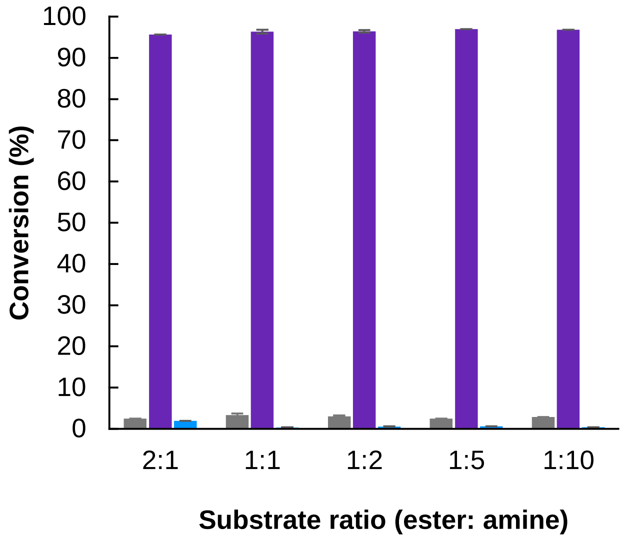

Figure S3–Varying amine concentration for no enzyme control: N-acylation of benzylamine with methyl furoate. Conditions: 10 mM ester, 200 mM Tris buffer pH 9, 25°C, 200 rpm, 18 h.

2.3 Model reaction optimization- enzyme loading

Table S3- Varying enzyme concentration for PestE catalysed N-acylation of benzylamine with methyl furoate. Conditions: 10 mM ester, 20 mM amine HCl, 200mM Tris buffer pH 9, 25°C, 200 rpm, 18 h.

[Enzyme] (μM)	% Acid **4**	% Ester **1**	% Amide **3**
0	4	96	-
1	9	68	23
5	11	31	59
10	13	17	70
50	21	-	79
100	14	-	87

2.4 Model reaction optimization- ester concentration

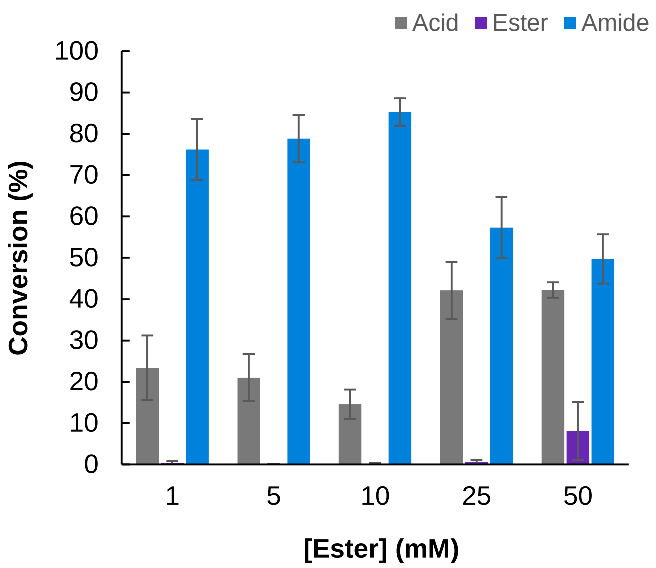

Figure S4- Varying ester concentration for PestE catalysed N-acylation of benzylamine with methyl furoate. Conditions: 100 μM PestE, 20 mM amine HCl, 200mM Tris buffer pH 9, 25°C, 200 rpm, 18 h.

2.5 Model reaction optimization- pH

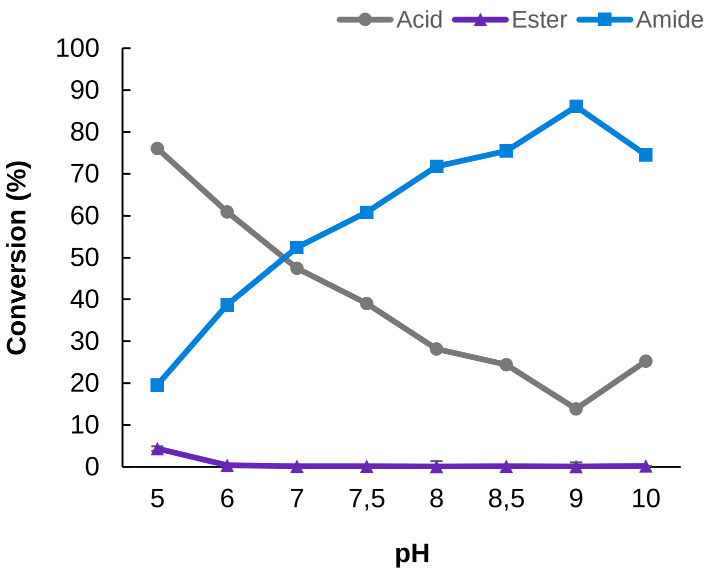

Figure S5- Varying pH for PestE catalysed N-acylation of benzylamine with methyl furoate. Conditions: 100 μM PestE, 10 mM ester, 20 mM amine HCl, 25°C, 200 rpm, 18 h.

2.6 Model reaction optimization- temperature

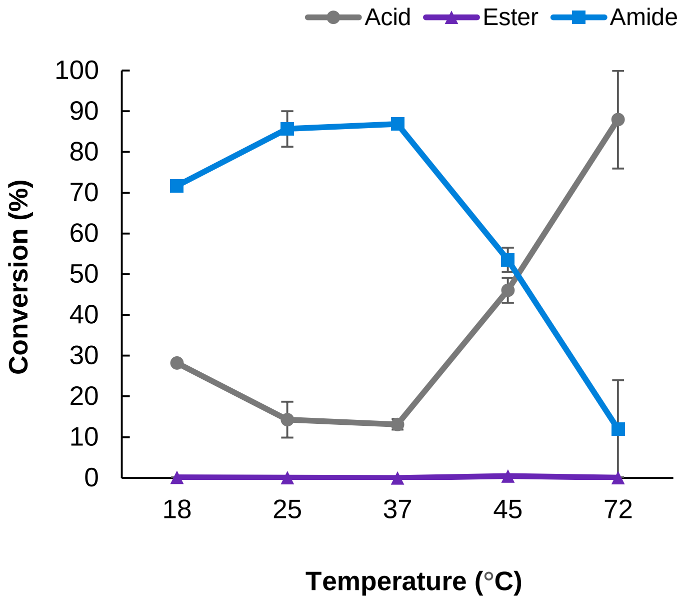

Figure S6- Varying temperature for PestE catalysed N-acylation of benzylamine with methyl furoate. Conditions: 100 µM PestE, 10 mM ester, 20 mM amine HCl, 200 mM Tris buffer pH 9, 200 rpm, 18 h.

2.7 Reaction conversions for PestE catalysed amide formation- substrates not accepted

Table S4- PestE catalysed amide formation. Conditions: 100 µM PestE, 10 mM ester, 100 mM amine HCl, 200 mM Tris buffer pH 9, 25°C, 200 rpm, 18 h.

Substrate	Acid % conversion **4**	Ester % **1**	Amide % conversion **X**
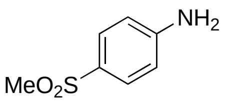	8	92	-
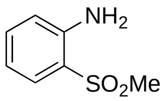	33	67	-
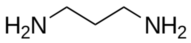	>99	-	-
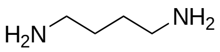	99	-	1
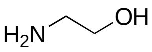	97	3	-

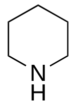	98	2	-
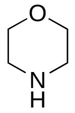	72	28	-
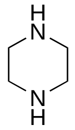	>99	-	-
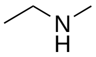	97	3	-

2.8 PestE catalysed tamibarotene formation- reaction optimization.

Table S5- PestE catalysed aminolysis of MMT **54** with amine **92** to Tamibarotene **93**. Conditions: 100 μM PestE, NaPi buffer pH 8, 25 °C, 250 rpm, 18 h. Varying substrate concentration as specified in table.

[mmTA] (mM)	[amine] (mM)	% Acid **71**	% Ester **54**	% Amide **93**
1	2	86	12	3
5	5	90	7	3
“	10	63	15	23
“	50	74	8	18
10	20	91	6	3

Table S6- pH screen for PestE catalysed aminolysis of MMT **54** with amine **92** to Tamibarotene **93**. Conditions: 100 μM PestE, 5 mM mmTA, 10 mM benzylamine HCl, 25 °C, 250 rpm, 18 h.

		% Acid **71**	% Ester **54**	% Amide **93**
	7	80	11	9
	8	81	2	17
pH	9	83	1	16

	10	42	47	10
Temperature	25	46	34	20
	37	99	0.6	0.4
	42	76	24	0.4

2.9 PestE catalysed amide formation from MHET and benzylamine.

Table S7- PestE catalysed formation of amides from MHET **91**. Conditions: 100 μM PestE, 5 mM MHET, 10 mM amine HCl, NaPi buffer pH 8, 25 °C, 250 rpm, 18 h.

Amine substrate	% Acid **71**	% Ester **91**	% Amide **88**
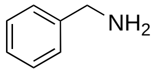	46	29	25 (5)

2.10 Terephthalic acid to amides cascade catalysed by FtpM and PestE.

Table S8- 2-step enzymatic cascade from terephthalic acid to amides. Conditions: 500 μM FtpM, 5 mM TA, 10 mM SAM, 20 μM SAH-nuc, pH 8, 25° C, 250 rpm, 18h followed by addition of100 μM PestE and 50 mM amine for a further 18 h.

Amide product	Acid (% conv.) (**71**)	Ester (%) (**54**)	Amide (% conv.) (**X**)
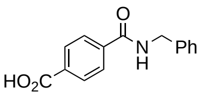 **88**	58	26	16
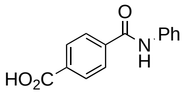 **94**	38	53	8
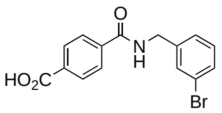 **95**	65	19	16
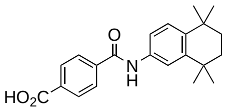 **93**	69	19	12

2.11 Time course reactions for all PestE mutants.

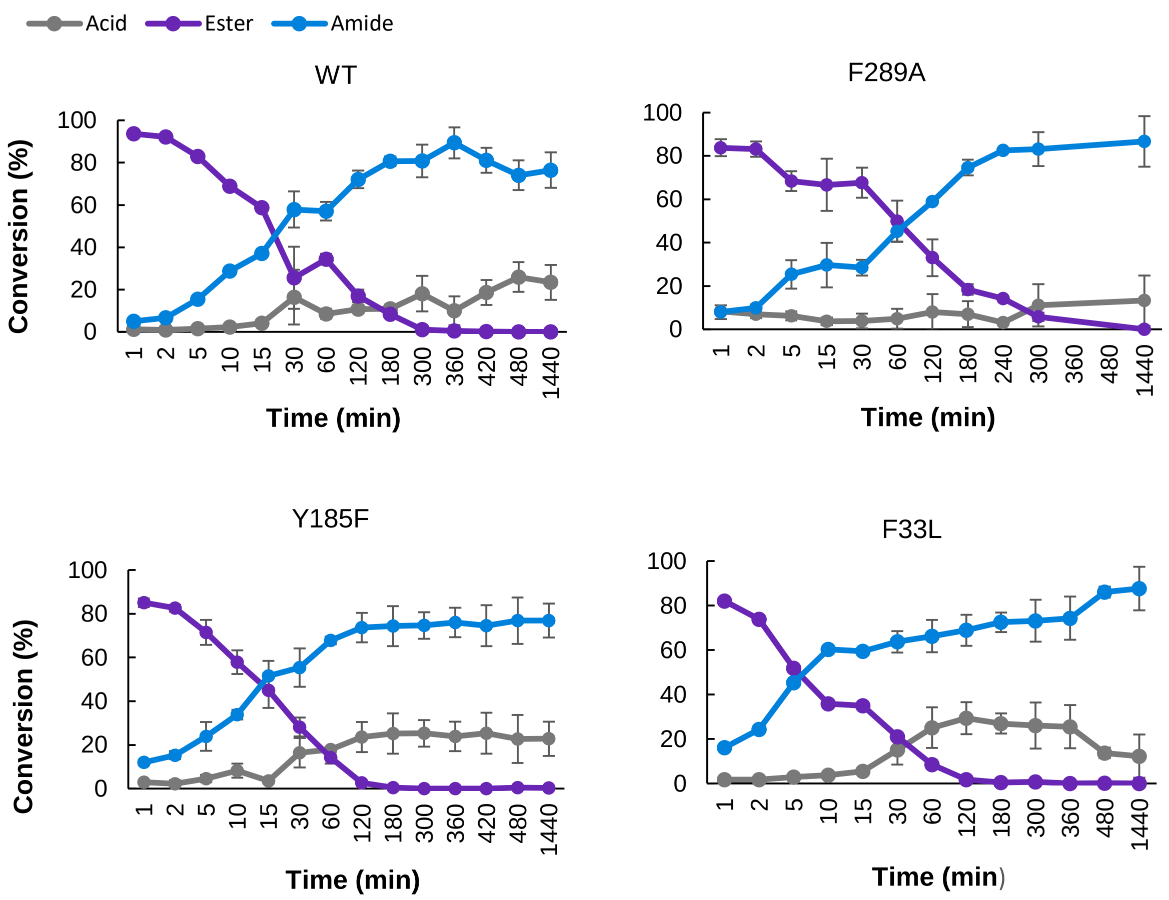

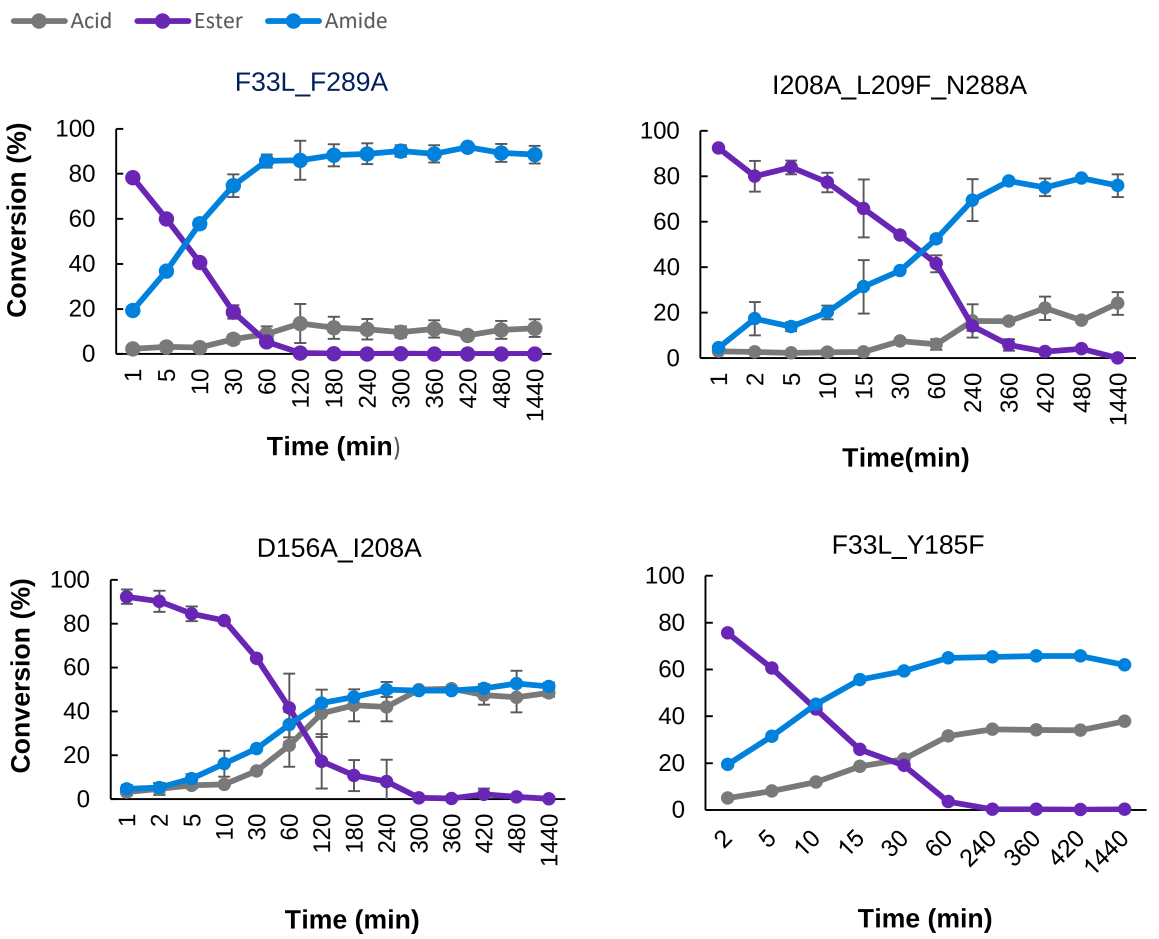

Figure S7- Time course for N-benzylfuranamide **3** formation catalysed by PestE mutants. Conditions: 100 μM PestE mutant, 10 mM methyl 2-furoate, 10 mM benzylamine HCl, Tris buffer pH 9, 25°C, 250 rpm, 18 h.

2.12 Representative chromatograms.

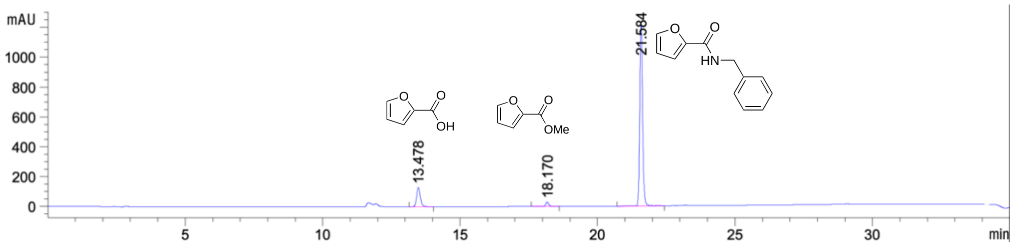

Figure S8- PestE (100 μM) catalysed formation of N-benzylfuranamide with methyl 2-furoate (10 mM) and benzylamine HCl (100 mM) at pH 9, 25 C, 18 h. Measured at 254 nm.

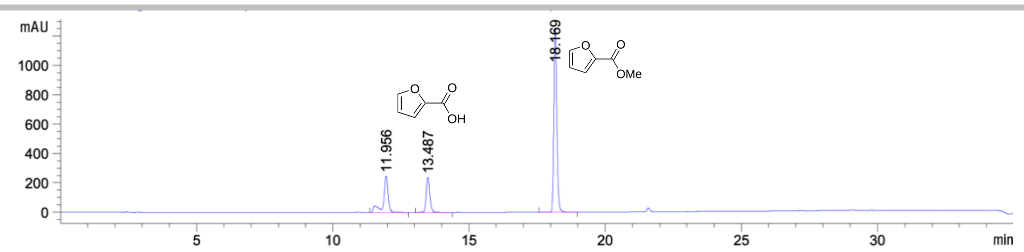

Figure S9- Control reaction of methyl 2-furoate (10 mM) and benzylamine HCl (100 mM) at pH 9, 25 C, 18 h (in the absence of PestE). Measured at 254 nm.

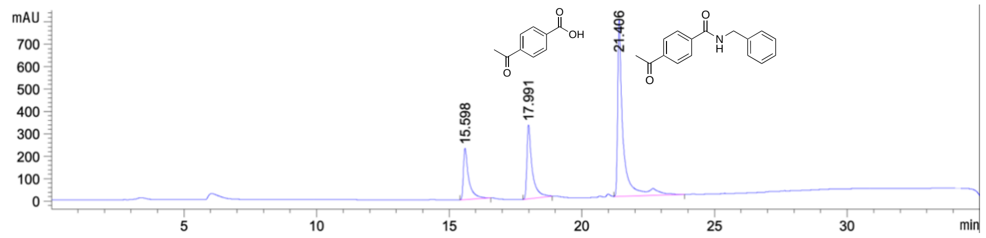

Figure S10- PestE (100 μM) catalysed formation of with methyl 4-acetyl benzoate (10 mM) and benzylamine HCl (100 mM) at pH 9, 25 C, 18 h. Measured at 240 nm. Caffeine as internal standard is at 15.7 min.

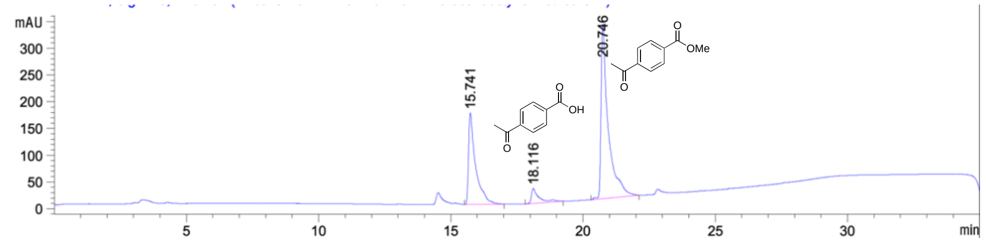

Figure S11- Control reaction of methyl 4-acetyl benzoate (10 mM) and benzylamine HCl (100 mM) at pH 9, 25 C, 18 h (in the absence of PestE). Measured at 240 nm. . Caffeine as internal standard is at 15.7 min.

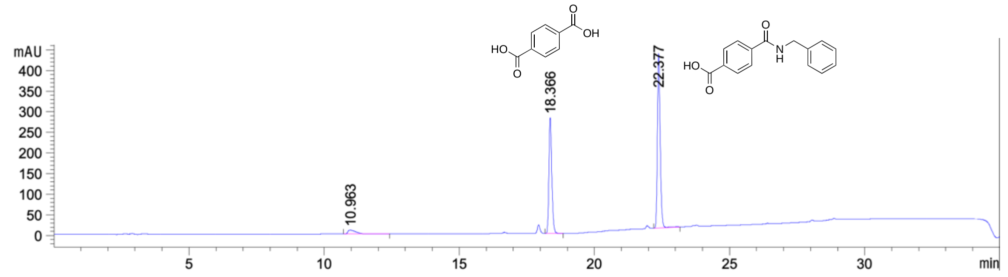

Figure S12- PestE (100 μM) catalysed formation of monomethyl terephthalate (10 mM) and benzylamine HCl (100 mM) at pH 9, 25 C, 18 h. Measured at 240 nm.

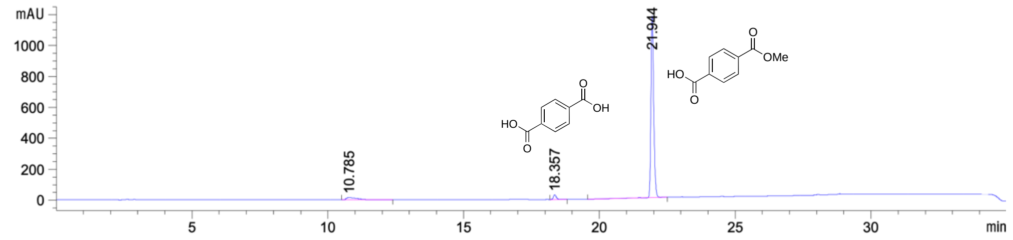

Figure S13-Control reaction of monomethyl terephthalate (10 mM) and benzylamine HCl (100 mM) at pH 9, 25 C, 18 h (in the absence of PestE). Measured at 240 nm.

References

1. L. C. Ward, H. V. McCue, D. J. Rigden, N. M. Kershaw, C. Ashbrook, H. Hatton, E. Goulding, J. R. Johnson and A. J. Carnell, *Angew Chem Int Ed*, 2022, **61**, e202117324

2. J. C. Sadler and S. Wallace, *Green Chemistry*, 2021, **23**, 4665–4672.

3. D. Parisi, C. Riley, A. S. Srivastava, H. V. McCue, J. R. Johnson and A. J. Carnell, *Green Chem*, 2019, **21**, 3827–3833

4. P. Ye, Y. Shao, X. Ye, F. Zhang, R. Li, J. Sun, B. Xu and J. Chen, *Org.Lett*, 2020, **22**, 1306–1310.

5. C. Zhu, W. Wei, P. Du and X. Wan, *Tetrahedron*, 2014, **70**, 9615–9620.
6. Y. Jiang, X. Li, X. Wang, Z.Wang, J. Zhang, J. Wu, W. Xu, *Chem Biol Drug Des*, 2016, **88**, 542-555

7. J. Eberhardt, D. Santos-Martins, A. F. Tillack and S. Forli, *J. Chem. Inf. Model*, 2021, **61**, 3891–3898

Author Contributions

AJC devised the project and acquired funding. AJC and JESS supervised the project. EG expressed PestE, performed substrate docking to identify PestE mutants (and expressed), characterized enzyme activity, performed reaction optimization, substrate scope and cascade from terephthalic acid to amides. Also, synthesis of standards. LCW expressed and characterized FtpM and alongside FEA, performed the PET hydrolysis and PET to MMT cascade. FEA also performed the preparative scale biotransformations (*N*-benzylfuranamide and tamibarotene). DD aided with PestE substrate scope analysis and synthesis of standards. EG and AJC wrote the manuscript and generated the figures.

NMR Spectra (next page)

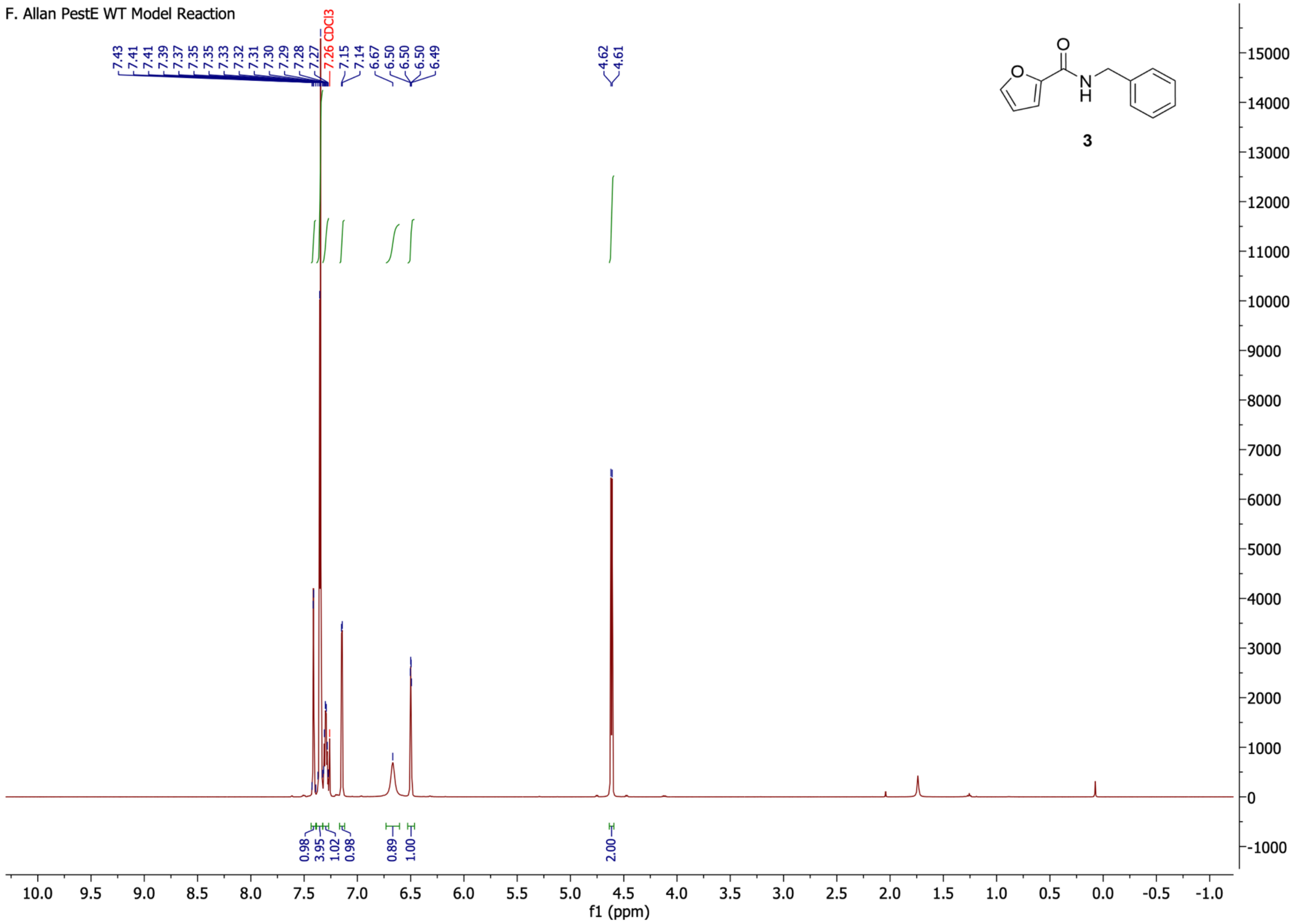
F. Allan PestE WT Model Reaction
7.43
7.41
7.41
7.39
7.37
7.35
7.35
7.33
7.32
7.31
7.30
7.29
7.28
7.27
7.26 CDCl3
7.15
7.14
6.67
6.50
6.50
6.50
6.49
4.62
4.61
O
O
N
H
3
0.98
3.95
1.02
0.98
0.89
1.00
2.00
f1 (ppm)

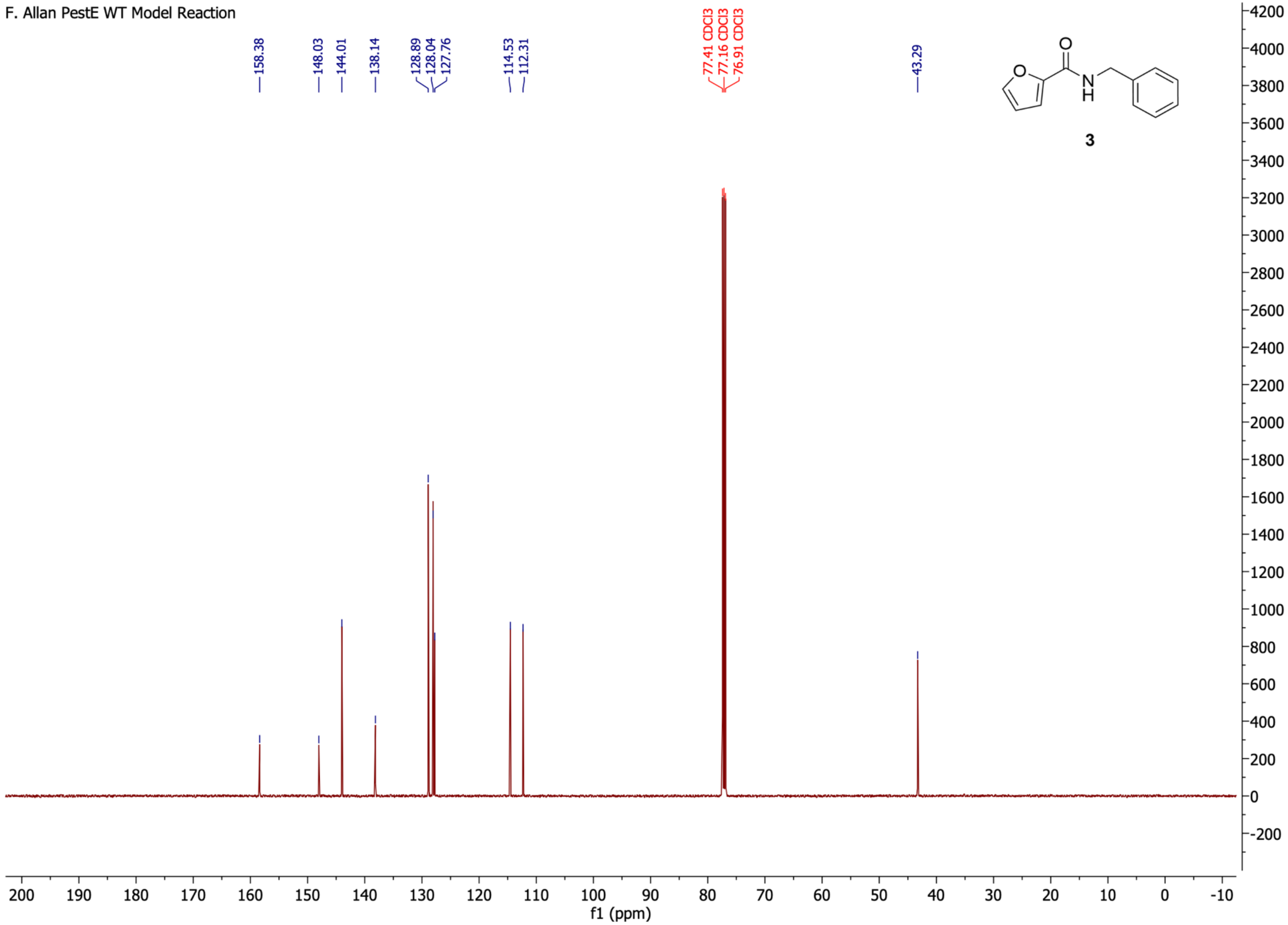
F. Allan PestE WT Model Reaction
158.38
148.03
144.01
138.14
128.89
128.04
127.76
114.53
112.31
77.41 CDCl3
77.16 CDCl3
76.91 CDCl3
43.29
3
f1 (ppm)

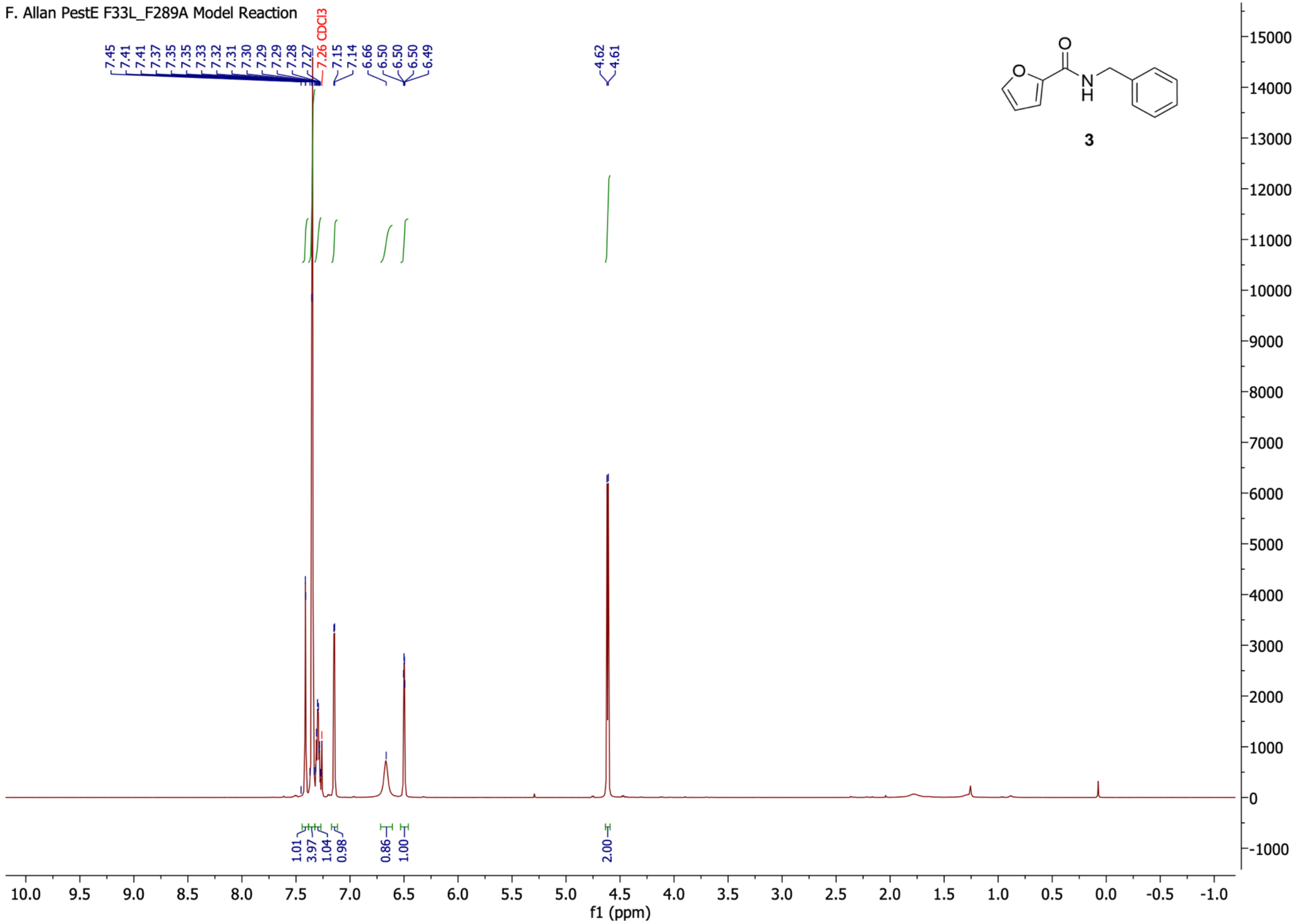
F. Allan PestE F33L_F289A Model Reaction
7.45
7.41
7.41
7.37
7.35
7.35
7.33
7.32
7.31
7.30
7.29
7.29
7.28
7.27
7.26 CDCl3
7.15
7.14
6.66
6.50
6.50
6.50
6.49
4.62
4.61
O
O
N
H
3
1.01
3.97
1.04
0.98
0.86
1.00
2.00
15000
14000
13000
12000
11000
10000
9000
8000
7000
6000
5000
4000
3000
2000
1000
0
-1000
10.0
9.5
9.0
8.5
8.0
7.5
7.0
6.5
6.0
5.5
5.0
4.5
4.0
3.5
3.0
2.5
2.0
1.5
1.0
0.5
0.0
-0.5
-1.0
f1 (ppm)

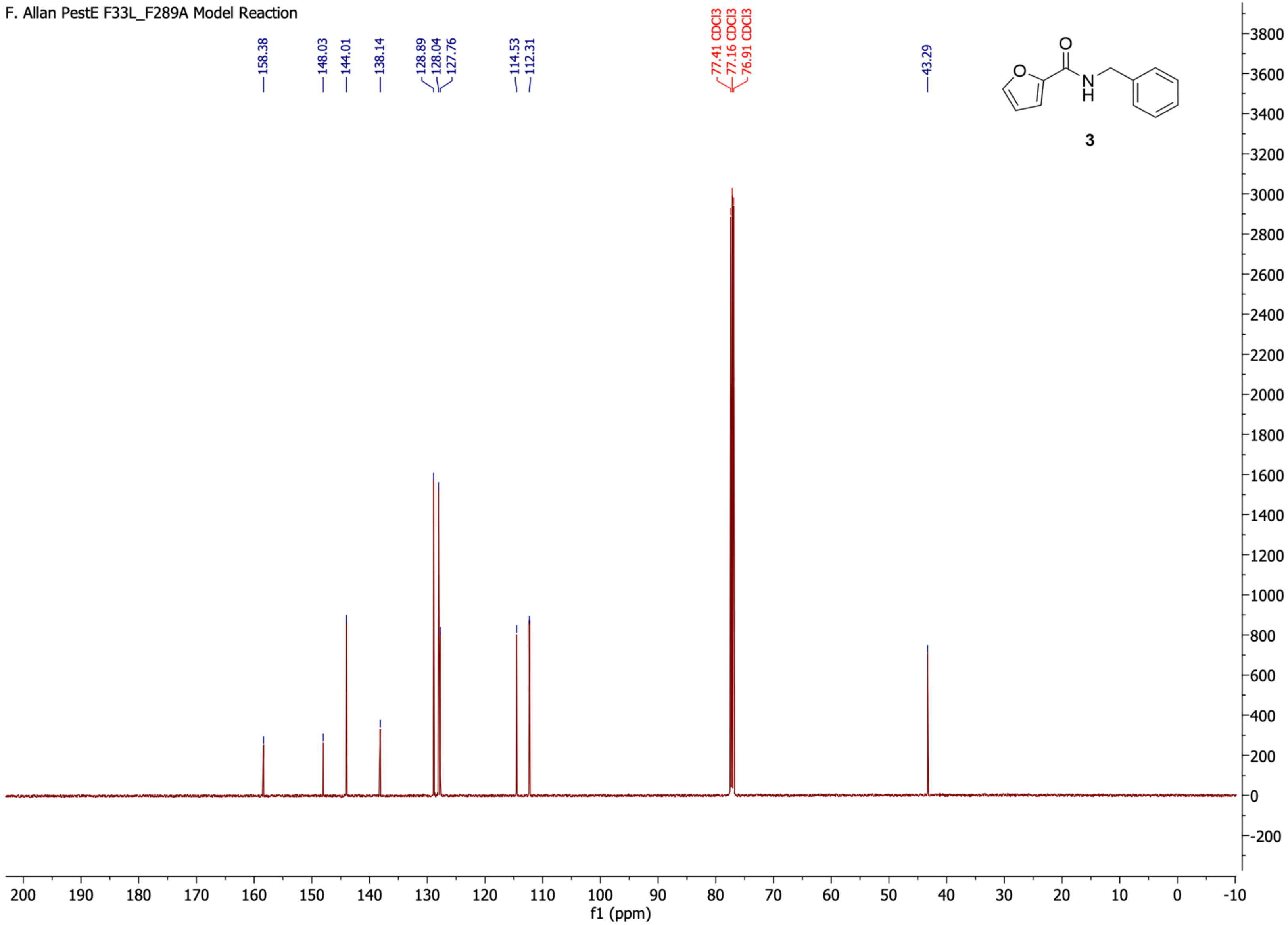
F. Allan PestE F33L_F289A Model Reaction
158.38
148.03
144.01
138.14
128.89
128.04
127.76
114.53
112.31
77.41 CDCl3
77.16 CDCl3
76.91 CDCl3
43.29
O
O
N
H
3
3800
3600
3400
3200
3000
2800
2600
2400
2200
2000
1800
1600
1400
1200
1000
800
600
400
200
0
-200
200 190 180 170 160 150 140 130 120 110 100 90 80 70 60 50 40 30 20 10 0 -10
f1 (ppm)

PestE F33A Tamibarotene

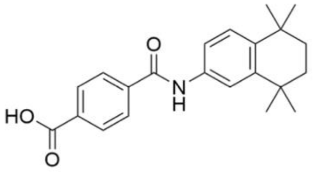

93

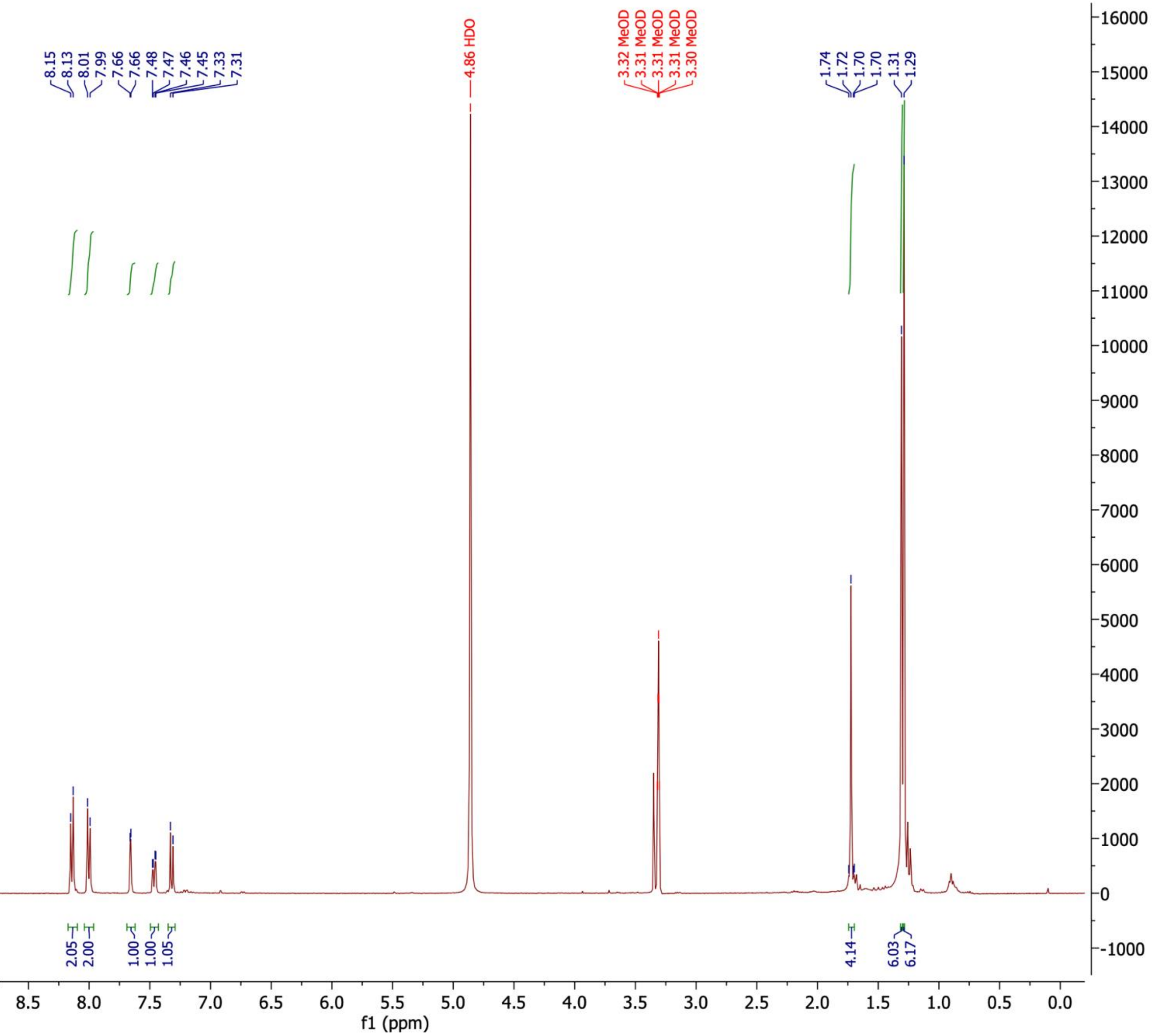

PestE F33A Tamibarotene

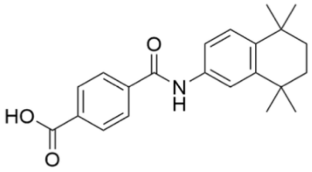

93

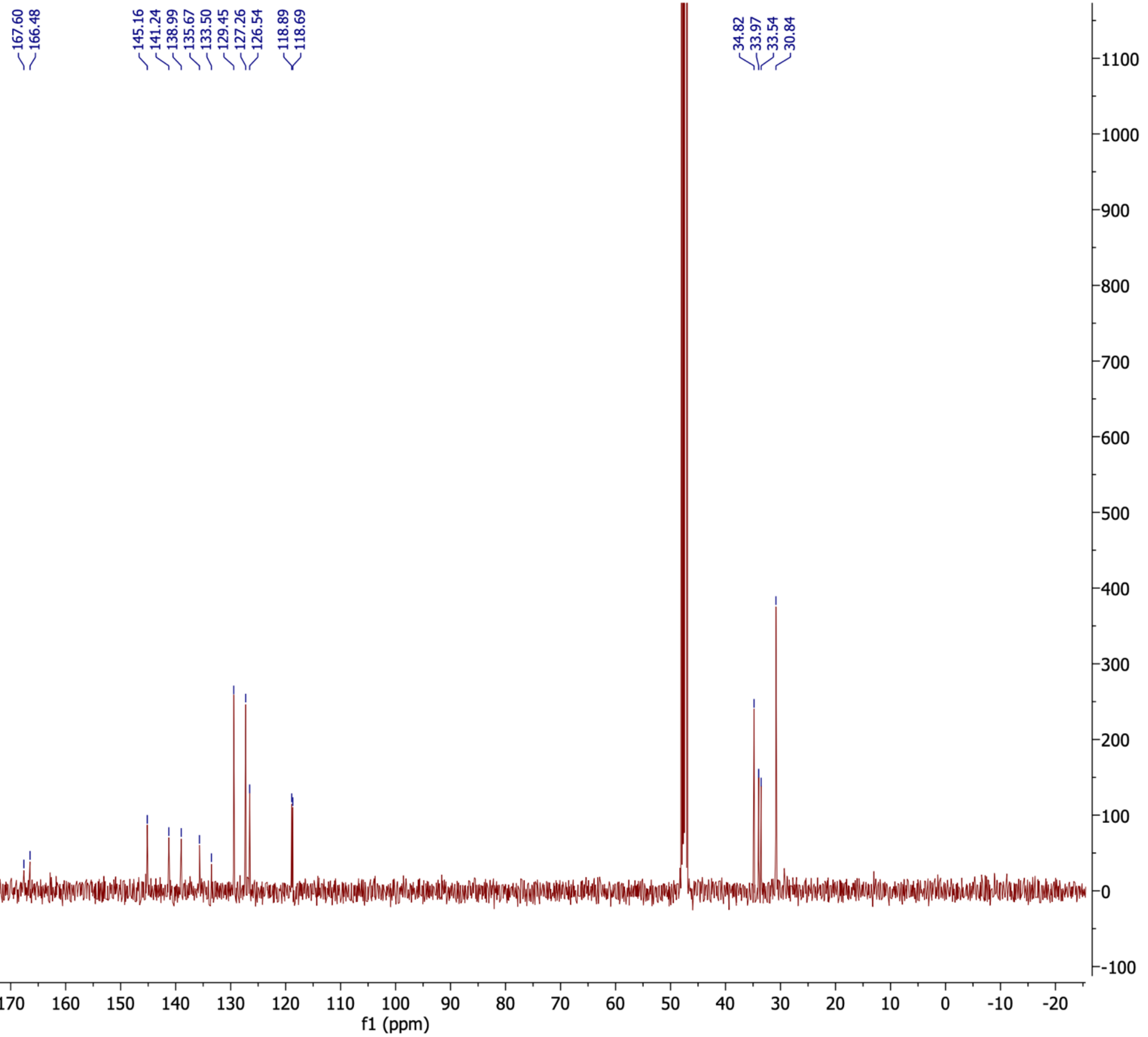

Supplement: Supplementary file 1 — Supporting Information [file ANIE-64-e202414162-s001.pdf]
